# Sequence-structure functional implications and molecular simulation of high deleterious nonsynonymous substitutions in *IDH1* revealed the mechanism of drug resistance in glioma

**DOI:** 10.3389/fphar.2022.927570

**Published:** 2022-09-16

**Authors:** Muhammad Suleman, Syeda Umme-I-Hani, Muhammad Salman, Mohammed Aljuaid, Abbas Khan, Arshad Iqbal, Zahid Hussain, Syed Shujait Ali, Liaqat Ali, Hassan Sher, Yasir Waheed, Dong-Qing Wei

**Affiliations:** ^1^ Centre for Biotechnology and Microbiology, University of Swat, Swat, Khyber Pakhtunkhwa, Pakistan; ^2^ Punjab Medical College, Faisalabad, Punjab, Pakistan; ^3^ Rashid Latif Medical College, Lahore, Punjab, Pakistan; ^4^ Department of Health Administration, College of Business Administration, King Saud University, Riyadh, Saudi Arabia; ^5^ Department of Bioinformatics and Biological Statistics, School of Life Sciences and Biotechnology, Shanghai Jiao Tong University, Shanghai, China; ^6^ Zhongjing Research and Industrialization Institute of Chinese Medicine, Zhongguancun Scientific Park, Meixi, Henan, China; ^7^ Division of Biology, Kansas State University, Manhattan, KS, United States.; ^8^ Centre for Plant Science and Biodiversity, University of Swat, Charbagh, Pakistan; ^9^ Office of Research, Innovation and Commercialization, Shaheed Zulfiqar Ali Bhutto Medical University (SZABMU), Islamabad, Pakistan; ^10^ State Key Laboratory of Microbial Metabolism, Shanghai-Islamabad-Belgrade Joint Innovation Center on Antibacterial Resistances, Joint Laboratory of International Cooperation in Metabolic and Developmental Sciences, Ministry of Education and School of Life Sciences and Biotechnology, Shanghai Jiao Tong University, Shanghai, China; ^11^ Peng Cheng Laboratory, Shenzhen, Guangdong, China

**Keywords:** nsSNPs, IDH1, molecular docking, simulation, binding free energy, introduction

## Abstract

In the past few years, various somatic point mutations of isocitrate dehydrogenase (IDH) encoding genes (IDH1 and IDH2) have been identified in a broad range of cancers, including glioma. Despite the important function of *IDH1* in tumorigenesis and its very polymorphic nature, it is not yet clear how different nsSNPs affect the structure and function of IDH1. In the present study, we employed different machine learning algorithms to screen nsSNPs in the *IDH1* gene that are highly deleterious. From a total of 207 SNPs, all of the servers classified 80 mutations as deleterious. Among the 80 deleterious mutations, 14 were reported to be highly destabilizing using structure-based prediction methods. Three highly destabilizing mutations G15E, W92G, and I333S were further subjected to molecular docking and simulation validation. The docking results and molecular simulation analysis further displayed variation in dynamics features. The results from molecular docking and binding free energy demonstrated reduced binding of the drug in contrast to the wild type. This, consequently, shows the impact of these deleterious substitutions on the binding of the small molecule. PCA (principal component analysis) and FEL (free energy landscape) analysis revealed that these mutations had caused different arrangements to bind small molecules than the wild type where the total internal motion is decreased, thus consequently producing minimal binding effects. This study is the first extensive *in silico* analysis of the *IDH1* gene that can narrow down the candidate mutations for further validation and targeting for therapeutic purposes.

## 1 Introduction

In primary brain tumor, glioblastoma, also known as grade IV glioma, is the most common and deadly form of brain tumor (Wirsching and Weller, 2017). In malignant gliomas, the primary GBMs account for 90% while the secondary GBMs that emerge from lower-grade gliomas (LGGs) in younger individuals account for less than 10% of clinical reports (Ohgaki and Kleihues, 2013). After the initial diagnosis, the survival for glioma individuals is from 14 to 16 months. Recent investigations revealed some metabolic features that are shared by virtually all GBMs and help to differentiate tumors from the normal brain (Tan et al., 2020). The GBM metabolic features are the excess generation of lactate in conjunction with the acetate and glucose oxidations to provide macromolecular precursors and energy. In low-grade glioma, secondary glioblastoma, and acute myelogenous leukemia, the oncogenic mutations in the two-isocitrate dehydrogenase (IDH) encoding genes (IDH1 and IDH2) have been identified (Agnihotri et al., 2013; Zhou and Wahl, 2019).

Normally in the Krebs cycle, the isocitrate is converted into a-ketoglutarate (a-KD) by isocitrate dehydrogenases (IDHs) in a NAD(P)-dependent manner. IDH1, IDH2, and IDH3 are three IDH isozymes that function in different subcellular compartments. Various somatic point mutations of IDH1 or IDH2 have been discovered in a variety of malignancies in recent years, such as gliomas and AMLs (acute myeloid leukemias) (Yan et al., 2009; Zhao et al., 2009). Identified mutations such as IDH1 R132H/C/Q, IDH2 R140Q/W/L, and R172K/T/S/G/M adversely affect the normal function of IDH protein and initiate the abnormal activity of protein with IDH mutations that produced oncometabolite 2-hydroxyglutarate from the a-KG (Frezza et al., 2010; Huang, 2019). 2-HG may accumulate to horrifically high levels of 5–35 mmol/g in human glioma samples with IDH1/2 mutations, which is 100-fold higher than its normal level in the brain (Dimitrov et al., 2015).

Single nucleotide polymorphisms (SNPs), which affect both coding and noncoding regions of DNA, are the most common genetic changes. SNPs are found every 200–300 bp in the human genome and account for around 90% of all genetic differences in the human genome. Nonsynonymous SNPs cause genetic alteration in the exonic regions of the protein and disturb their sequence, structure, and normal function by enhancing the abnormal transcription and translation mechanisms. Previously, several *in silico* computational techniques were developed to quickly and precisely assess the functional implications of nonsynonymous variation on protein structure and function (Junaid et al., 2018; Khan et al., 2020a; Khan et al., 2020b; Khan et al., 2021). Until now, a total of 298 SNPs with 207 missense mutations in the human IDH1 gene has been described and deposited to the gnomAD database.

Although IDH1 plays a crucial role in carcinogenesis (gliomas) and has a polymorphism character, it is still unclear how identified nsSNPs alter the protein’s structure and biological activity. In this study, we employed a number of computational approaches to find nsSNPs in the IDH1 gene that are extremely detrimental to the structure and function of the IDH1 protein.

## 2 Materials and methods

### 2.1 Collection of data

**FIGURE 1:**
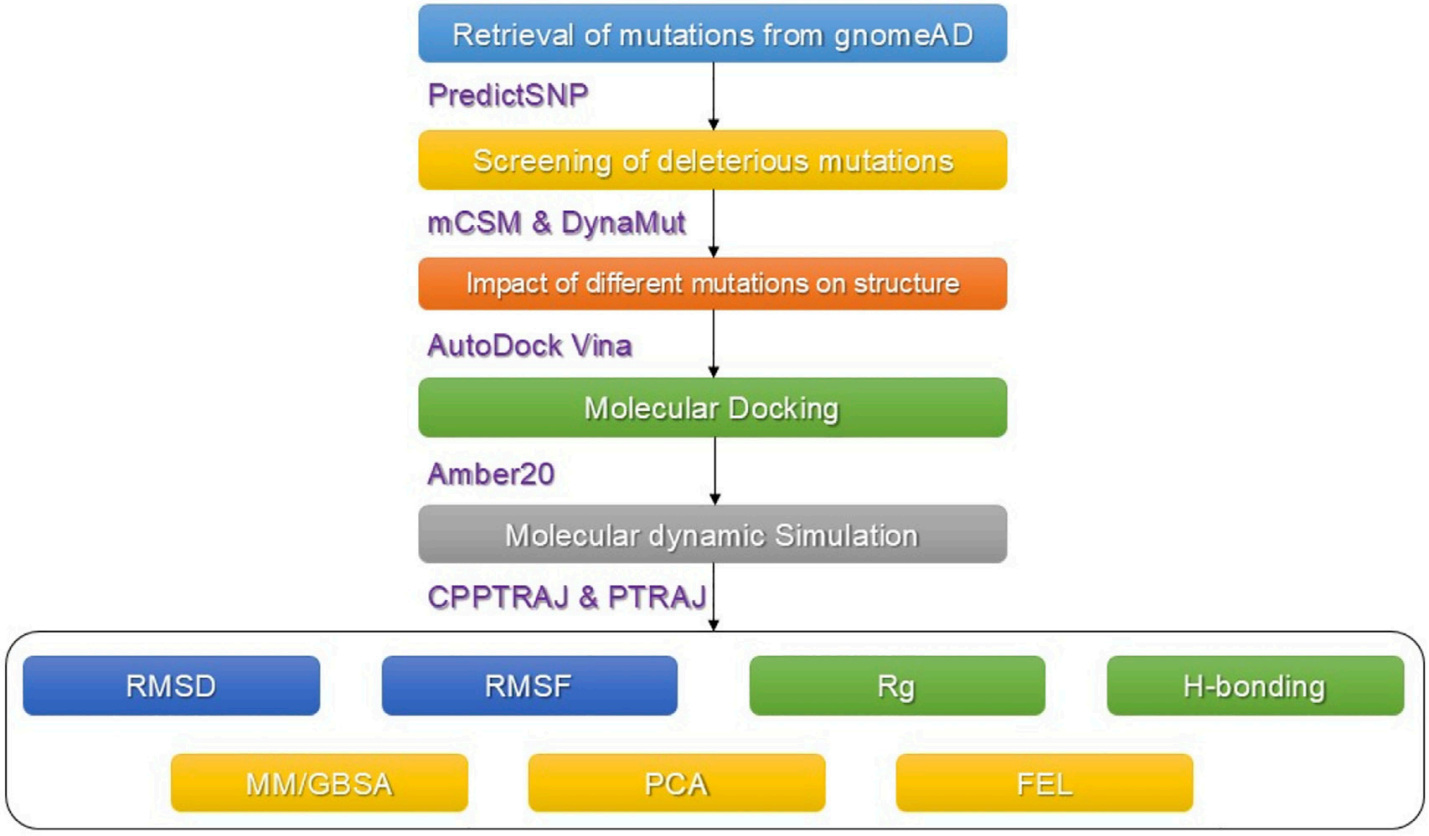
Methodological workflow of the work. Each tool used in each step is also given.

The available data on human IDH1 were obtained from available online databases. The online database gnomAD (https://gnomad.broadinstitute.org/) was used to retrieve all predicted SNPs in the human *IDH1*gene (Karczewski and Francioli, 2017). The amino acid sequence (UniProt: O75874) and previously deposited 3D structure (PDB ID: 6BKX) of the protein that expresses the *IDH1* gene were obtained from the UniProt online database (http://www.uniprot.org/) (Rose et al., 2010; Consortium, 2015). The whole workflow of the work is given in Figure 1.

### 2.2 Disease-related single nucleotide polymorphism predictions

#### 2.2.1 Prediction of Functional Consequences of nsSNPs

Different online servers such as PredictSNP (https://loschmidt.chemi.muni.cz/predictsnp1/), Polymorphism Phenotyping version 2 (Polyphen-2) (http://genetics.bwh.harvard.edu/pp2), Sorting Intolerant from Tolerant (SIFT) (http://sift.bii.a-star.edu.sg), Screening of nonacceptable polymorphism (SNAP) (https://rostlab.org/services/snap), Protein Analysis Through Evolutionary Relationship (PANTHER) (http://www.pantherdb.org/tools/csnpScoreForm.jsp, Multivariate Analysis of Protein Polymorphism (MAPP) (http://mendel.stanford.edu/SidowLab/downloads/MAPP/), and predictor of Human Deleterious Single Nucleotide Polymorphism (PhD-SNP) (http://snps.biofold.org/phd-snp/phd-snp.html) were used to predict the functional effect of all nonsynonymous single nucleotide polymorphisms (nsSNPs) (Johnson et al., 2008; Sim et al., 2012; Adzhubei et al., 2013; Landis et al., 2014; Bendl et al., 2015; Capriotti and Fariselli, 2017). All of the nsSNPs that were verified as highly deleterious by all of the aforementioned web servers were selected for further analysis.

#### 2.2.2 Structure-based stability calculation

For structure-based stability prediction, mCSM and DynaMut webservers were used to estimate the impact of each substitution on the structural stability and flexibility (Pires et al., 2014; Rodrigues et al., 2018). The highly deleterious mutations were processed for the prediction of structure-based stability calculation. These servers use graph-based signatures to estimate the impact of each mutation on the protein’s structure. The top three mutations were selected based on the mCSM and DynaMut results together for further analysis.

#### 2.2.3 Modeling of mutants of *IDH1* protein

The crystal structure of the *IDH1* protein was extracted from PDB (Entry ID: 6BKX). Both ligands and water molecules were separated from the protein structure, and the Chimera software was used to minimize the wild-type structure of *IDH1* protein. Moreover, the predicted most deleterious mutations such as G15E, W92G, and I333S were modeled in the wild-type structure of *IDH1* protein using the Chimera software.

#### 2.2.4 Molecular docking of DWP with the wild type and mutant *IDH1*

The impact of selected substitutions on the binding of DWP ((6aS,7S,9R, 10aS)-7,10a-dimethyl-8-oxo-2-(phenylamino)-5,6,6a,7,8,9,10,10a-octahydrobenzo[h]quinazoline-9-carbonitrile with the wild type and mutant was also evaluated using the molecular docking approach. For this estimation, a previously described protocol was employed using AutoDock Vina (Eberhardt et al., 2021).

#### 2.2.5 Molecular dynamics simulation

The highly destabilizing and functional substitutions were evaluated for the dynamic properties using the AMBER2.0 molecular simulation tool. For this purpose, ff14SB force field was recruited for uniformity with the previous parameters (Salomon Ferrer et al., 2013). A TIP3P water box (cutoff = 10.0 Å) was employed for solvation, while neutralizations were performed by adding sodium ions. Each complex was minimized well in two steps: the first for 6000 steps, while the second was run for 3,000 steps. The further protocol used in the previous study was employed. Lastly, for each complex, a 100 ns production run under constant pressure was completed. To control the temperature, a Langevin thermostat with 1 atm pressure and 300 K was used (Zwanzig, 1973). The particle mesh Ewald (PME) algorithm was used to compute long-range interactions (Darden et al., 1993; Essmann et al., 1995). The cutoff distances were set to 10 Å. For the covalent bonds involving hydrogen, the SHAKE algorithm was used (Ryckaert et al., 1977). GPU-accelerated simulation (PMEMD.CUDA) was used for all of the processes. Post simulation analyses including dynamic stability calculated as RMSD (root mean square deviation), residual flexibility estimated as RMSF(root mean square fluctuation), hydrogen bonding analysis over the simulation time, and the radius of gyration (Rg) for protein packing assessment were calculated using CPPTRAJ and PTRAJ modules of AMBER1.9 (Roe and Cheatham, 2013).

#### 2.2.6 Binding free energy calculation

For the calculation of binding free energy, a whole trajectory of each complex was subjected to MM/GBSA analysis by utilizing MMPBSA.PY script (Hou et al., 2011). This widely applicable approach, which has been previously used to characterize the binding of various biological complexes, was used for estimation by employing the following equation:

ΔGbind=ΔGcomplex−[ΔGreceptor+ΔGligand]. (1)

Each term in the binding free energy was estimated using the following equation:

G=Gbond+Gele+GvdW+Gpol+Gnpol. (2)

#### 2.2.7 Clustering of MD trajectories using principal component analysis

To comprehend the motion of MD trajectories, an unsupervised learning method known as principal component analysis (PCA) (Pearson, 1901; Wold et al., 1987) was performed to acquire knowledge regarding the internal motion of the system. For this purpose, an Amber module known as CPPTRAJ was used. The spatial covariance matrix was determined for eigenvector and their atomic co-ordinates. Using an orthogonal coordinate transformation, a diagonal matrix of eigenvalues was generated. Based on the eigenvectors and eigenvalues, the principal components were extracted. Using these PCs, the dominant motions during simulation were plotted (Balsera et al., 1996; Ernst et al., 2015).

## 3 Results and discussion

### 3.1 Identification of deleterious nsSNPs

The online public resources were used to retrieve all of the available data on the human *IDH1* gene. According to the information obtained from the online gnomAD database, there were a total of 298 SNPs in the *IDH1* protein. Of these, 207 SNPs were identified as nonsynonymous. These 207 SNPs were submitted to a different online server to identify the deleterious mutations. First, the SNPs were submitted to PredictSNP and MAPP servers, and only 141 and 140 SNPs were found to be deleterious, respectively **(** Supplementary Table S1 **)**. The nsSNPs were then submitted to PhD-SNP and SNAP online tools and found 63 and 55 SNPs as deleterious, respectively (Supplementary Table S2). The other online servers such as PolyPhen-1, PolyPhen-2, SIFT, and PANTHER analyzed the nsSNPs and predicted that out of 119 SNPs only 51, 46, 68, and 80 were deleterious, respectively **(** Supplementary Tables S3, S4 **)**. All of the nsSNPs were selected for further analysis and was predicted as highly deleterious together by all of the abovementioned online servers. Only 80 mutations were selected for the structure-based stability analysis using mCSM as shown in Table 1. mCSM predicted that only 14 mutations (G15E(-3.09), W23C(-2.14), R49H(-2.43), I76T(-2.36), R82S(-2.36), W92G(-3.64), F108V(-2.44), V152G(-2.06), Y208H(-2.64), Y231H(-2.00), Y246H(-2.03), I330T(-2.44), I333S(-3.29), and I367T(-2.84)) out of 80 were highly destabilizing. These 14 mutations were further verified using the DynaMut web server.

**TABLE 1:** List of highly deleterious and destabilizing mutations in IDH1. Among the 80 mutations, 14 highly destabilizing ones are shown in bold.

Index	Mutation	ΔΔG mCSM	Outcome
1	**G15E**	**−3.094**	**Highly Destabilizing**
2	D16H	−1.439	Destabilizing
3	**W23C**	−**2.149**	**Highly Destabilizing**
4	Y34C	−1.848	Destabilizing
5	V35A	−1.926	Destabilizing
6	Y42C	−1.287	Destabilizing
7	D43A	−0.396	Destabilizing
8	**R49H**	−**2.437**	**Highly Destabilizing**
9	R49C	−1.957	Destabilizing
10	R49P	−1.427	Destabilizing
11	**I76T**	−**2.36**	**Highly Destabilizing**
12	**R82S**	−**2.362**	**Highly Destabilizing**
13	R82M	−1.294	Destabilizing
14	V83F	−1.491	Destabilizing
15	E85G	−1.128	Destabilizing
16	L88F	−1.714	Destabilizing
17	M91R	−0.476	Destabilizing
18	M91T	−1.317	Destabilizing
19	**W92G**	−**3.644**	**Highly Destabilizing**
20	W92R	−1.562	Destabilizing
21	N96H	−0.752	Destabilizing
22	T98N	−1.142	Destabilizing
23	N101Y	−0.377	Destabilizing
24	T106M	−0.611	Destabilizing
25	**F108V**	−**2.441**	**Highly Destabilizing**
26	R109K	−1.318	Destabilizing
27	I113S	−1.972	Destabilizing
28	G150R	−0.548	Destabilizing
29	**V152G**	−**2.065**	**Highly Destabilizing**
30	I154R	0.027	Stabilizing
31	D160Y	0.05	Stabilizing
32	G177D	−0.073	Destabilizing
33	Y183C	−0.838	Destabilizing
34	A193T	−0.975	Destabilizing
35	L207W	−1.744	Destabilizing
36	**Y208H**	−**2.648**	**Highly Destabilizing**
37	Y208C	−1.847	Destabilizing
38	T214S	−0.522	Destabilizing
39	Y219H	−0.962	Destabilizing
40	Y219C	−0.98	Destabilizing
41	D220G	−1.683	Destabilizing
42	**Y231H**	−**2.007**	**Highly Destabilizing**
43	Y235C	−1.24	Destabilizing
44	**Y246H**	−**2.036**	**Highly Destabilizing**
45	A256V	−0.53	Destabilizing
46	K260N	0.221	Stabilizing
47	G263E	−0.546	Destabilizing
48	D273G	−0.293	Destabilizing
49	G274S	−0.962	Destabilizing
50	V276M	−0.5	Destabilizing
51	S278P	−0.225	Destabilizing
52	S278L	−0.228	Destabilizing
53	D279H	−0.517	Destabilizing
54	M291T	−1.405	Destabilizing
55	T292I	−0.114	Destabilizing
56	S293I	0.169	Stabilizing
57	P298L	−0.358	Destabilizing
58	G300V	−0.604	Destabilizing
59	G300D	−1.749	Destabilizing
60	E306A	0.57	Stabilizing
61	H309R	−0.568	Destabilizing
62	H309Q	0.139	Stabilizing
63	G310R	−0.445	Destabilizing
64	R314C	0.301	Stabilizing
65	H315D	−0.52	Destabilizing
66	R317C	−0.995	Destabilizing
67	R317L	−0.082	Destabilizing
68	T325M	0.467	Stabilizing
69	N328S	−1.25	Destabilizing
70	N328K	−0.122	Destabilizing
71	**I330T**	**−2.449**	**Highly Destabilizing**
72	**I333S**	−**3.298**	**Highly Destabilizing**
73	G339R	−1.576	Destabilizing
74	L346P	−1.191	Destabilizing
75	**I367T**	−**2.845**	**Highly Destabilizing**
76	G370V	−0.276	Destabilizing
77	M372T	−1.565	Destabilizing
78	T373I	−0.732	Destabilizing
79	T373N	−1.561	Destabilizing
80	L401P	-1.626	Destabilizing

**FIGURE 2:**
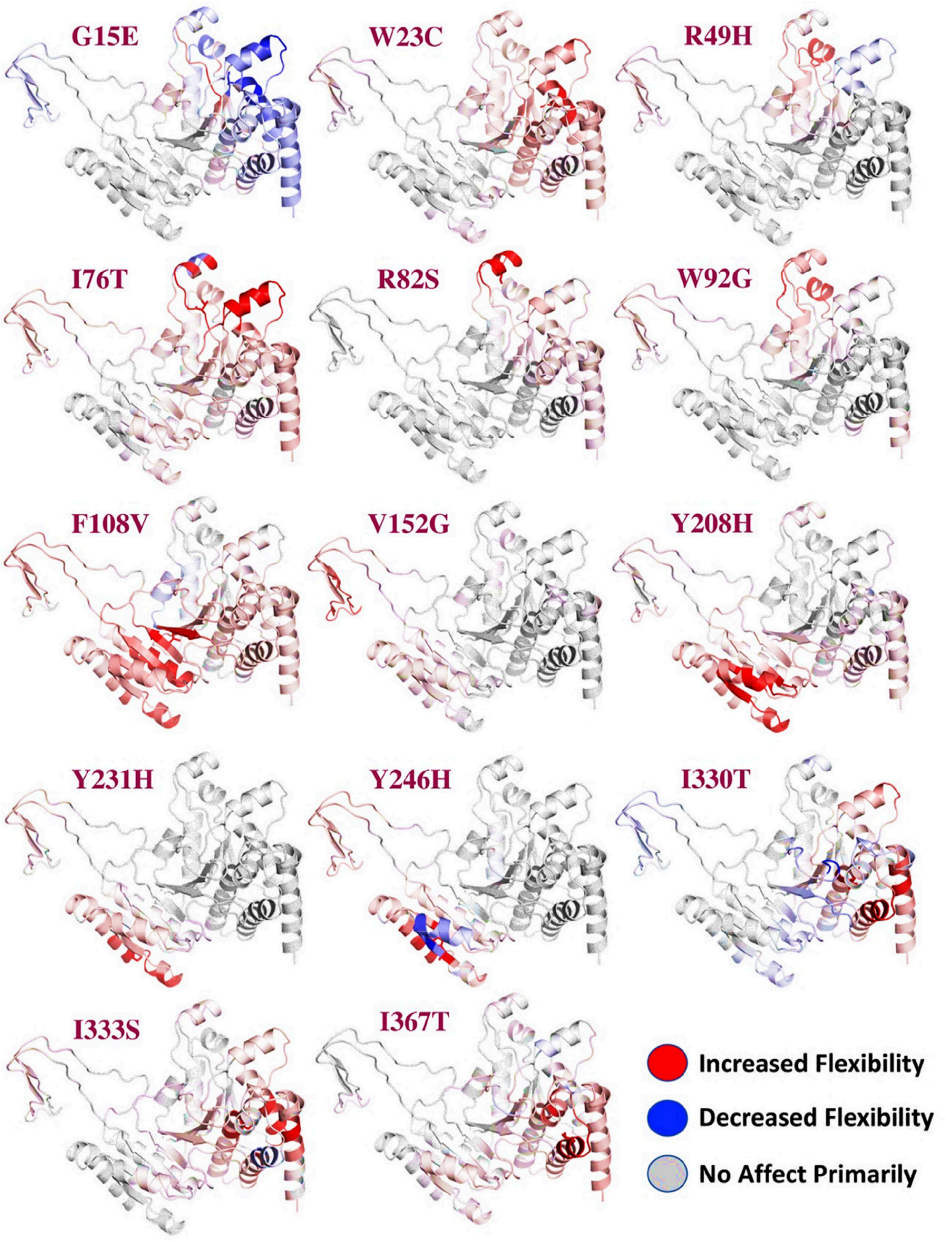
Effect of mutations on the flexibility of different residues. Different colors represent different levels of flexibility. The red regions demonstrate that the flexibility is increased, the blue regions show that the flexibility is decreased due to the mutations, and gray represents no change in the flexibility.

The reported 14 highly destabilizing mutations were then processed using the DynaMut server to determine the effect of these 14 mutations on the flexibility of protein structure. Among these 14 mutations, 12 mutations induced higher flexibility while the other two mutations, G15E and V246H, demonstrated structural rigidity. These changes in flexibility shown in red and blue were mapped onto the corresponding protein structure and are presented in Figure 2. Among these 14 mutations, only three mutations were reported to be consistently highly destabilizing, which were then selected for further analysis (Table 2).

**TABLE 2:** List of highly deleterious and destabilizing mutations in IDH1. Among the 14 mutations, three are highly destabilizing reported by both the servers (mCSM and DynaMut) shown in bold.

Index	Mutation	ΔΔG ENCoM	ΔΔG DynaMut
1	**G15E**	**1.101**	**−3.347**
2	W23C	−0.977	−0.288
3	R49H	−0.348	−1.701
4	I76T	−0.198	−1.926
5	R82S	−0.607	−2.139
6	**W92G**	−**1.741**	−**2.633**
7	F108V	−0.341	−2.520
8	V152G	−0.894	−1.667
9	Y208H	−0.662	−0.591
10	Y231H	−0.369	−0.945
11	Y246H	−0.136	−0.100
12	I330T	−0.171	−0.733
13	**I333S**	−**0.478**	−**3.413**
14	I367T	−0.318	−2.610

### 3.2 Molecular docking analysis of the wild type and mutants

**FIGURE 3:**
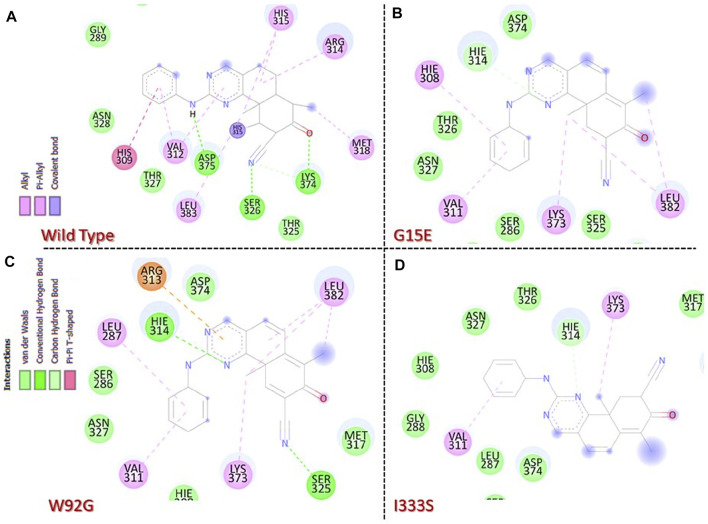
Molecular docking analysis of the wild-type and mutant complexes. **(A**) Representation of the interaction pattern of the wild type. **(B)** Representation of the interaction pattern of G15E. **(C)** Representation of the interaction pattern of W92G. **(D)** Representation of the interaction pattern of I333S.

Molecular docking-based investigation of the binding variations caused by these mutations in contrast to the wild type revealed significant differences. The docking score for the wild type was calculated to be −8.76 kcal/mol. The interaction analysis revealed multiple hydrogen bonds including Ser326, Lys374, and Asp375, while various pi–pi interactions and pi–alkyl interactions were observed. The interaction pattern of the wild type is given in Figure 3A. On the other hand, despite the significant reduction in the number of bonding contacts, the His314 (correspond to His315) hydrogen bond remained conserved, which has been reported to be associated with the inhibitory properties of this drug. With a single hydrogen bond and various other interactions, the docking score for this complex was calculated to be −7.35 kcal/mol. It can be seen that this complex has lost important hydrogen bonds of essential residues, thus reducing the bonding energy and contributing to resistance to chemotherapy. The interaction pattern of G15E is given in Figure 3B. Moreover, the docking score for W92G was estimated to be −8.62 kcal/mol. This complex retained some important hydrogen contacts, that is, Ser326 and His314 (correspond to His315), which gives comparable results to the wild type. With various hydrogen bonding contacts, many pi–pi, pi–alkyl, and salt bridge contacts were also reported in this complex. The interaction pattern of W92G is given in Figure 3C. I333S lies near the active site and reports a substantial decrease in the bonding pattern. With only one hydrogen bond of His314 (corresponding to His315) and pi–alkyl interaction with Val311, this complex reported a significant decrease in the docking score. The docking score for this complex was calculated to be −6.87 kcal/mol. The interaction pattern of I333S is given in Figure 3D.

### 3.3 Investigation of the dynamic behavior of the wild-type and mutant complexes

**FIGURE 4:**
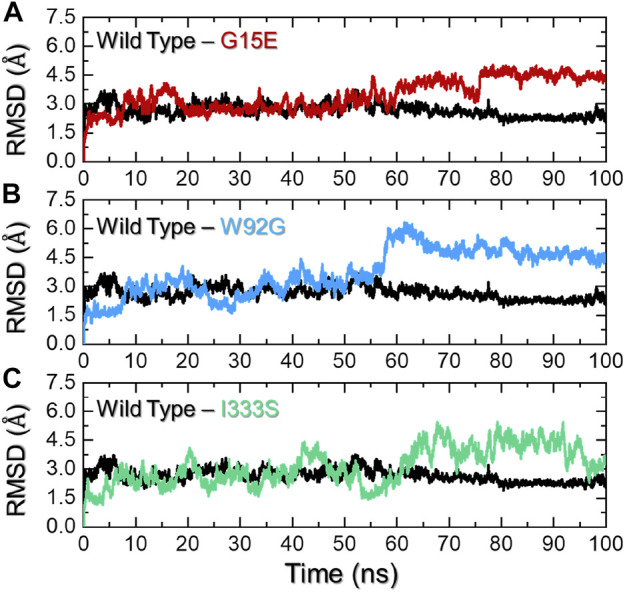
Dynamic stability analysis of the wild-type and mutant complexes**. (A**) Representation of the RMSD of the wild type and G15E. **(B)** Representation of the RMSD of the wild type and W92G. **(C)** Representation of the RMSD of the wild type and I333S. The x-axis represents time in nanoseconds while the y-axis represents RMSD in Å.

To provide worthy insights into the impact of any particular mutation on the structure and function of a protein comprehension of key dynamic features is essential. For instance, dynamic stability [root mean square deviation (RMSD)] can be used to estimate the stability of a biological complex in a dynamic environment. To assess the structural stability, we herein also calculated RMSD as a function of time. The wild type and the three mutants were compared and are shown in Figures 4A–C. The wild type presented more stable behavior than the three mutants. As shown in Figure 4A, the wild type initially demonstrated a higher RMD for a short period (2–6 ns); thereafter, the complex equilibrated and attained stability at 2.5 Å. The RMSD continued to follow the same trend until 55 ns and then decreased to 2.1 Å until the end of simulation. No significant perturbation was observed during the simulation, and the average RMSD was calculated to be 2.30 Å. Comparatively, the G15E mutant initially demonstrated significant perturbations in the RMSD, particularly between 5 and 20 ns. The RMSD then stabilized for a period between 21 and 55 ns and then continued to increase gradually until the end of the simulation. The complex reported a higher RMSD between 56 and 73 ns, then abruptly decreased, and then increased back with major deviation until the end of simulation. The complex reached the complete stability state after 80 ns. This shows that this mutation, despite its location away from the binding cavity, allosterically affects the binding affinity by compromising the stability of the protein. Since it has been previously reported that mutations that alter the protein stability result in radical function, thus this corroborates with the current findings (Dehury et al., 2020a). The RMSD graph for G15E mutant is given in Figure 4A. Unlike the wild type and G15E, the W92G complex demonstrated significant structural instability from the start of the simulation. The RMSD during the first 60 ns reported minor deviations at different time intervals, thus resulting in continuous destabilization effects of mutation. The RMSD increased to 6.0 Å at 60 ns and then gradually decreased until 100 ns. An average RMSD for W92G was recorded to be 3.60 Å and is shown in Figure 3B. On the other hand, the I333S mutant was reported to be the most destabilizing mutation among the shortlisted top deleterious mutations. First, the complex reported significant deviations until 55 ns and then gradually increased the RMSD and followed the same pattern to demonstrate significant deviations until the end of the simulation. An average RMSD of 3.2 Å was calculated for the I333S complex and is shown in Figure 4C. It has been reported that mutations that increase the stability may also increase the binding while destabilizing mutations decrease the binding. The current findings strongly corroborate with the previous reports where the filtration of mutations to obtain the most deleterious mutations showed that R132C, R132G, R132H, R132L, and R132S decrease the stability of IDH1 (Kumar et al., 2018). The stability feature is also reported to be affected by mutations in other diseases, which results in deleterious effects (Dehury et al., 2020a). Thus, herein, the mutations demonstrated destabilizing effects in contrast to the wild type.

### 3.4 Protein structure packing analysis

**FIGURE 5:**
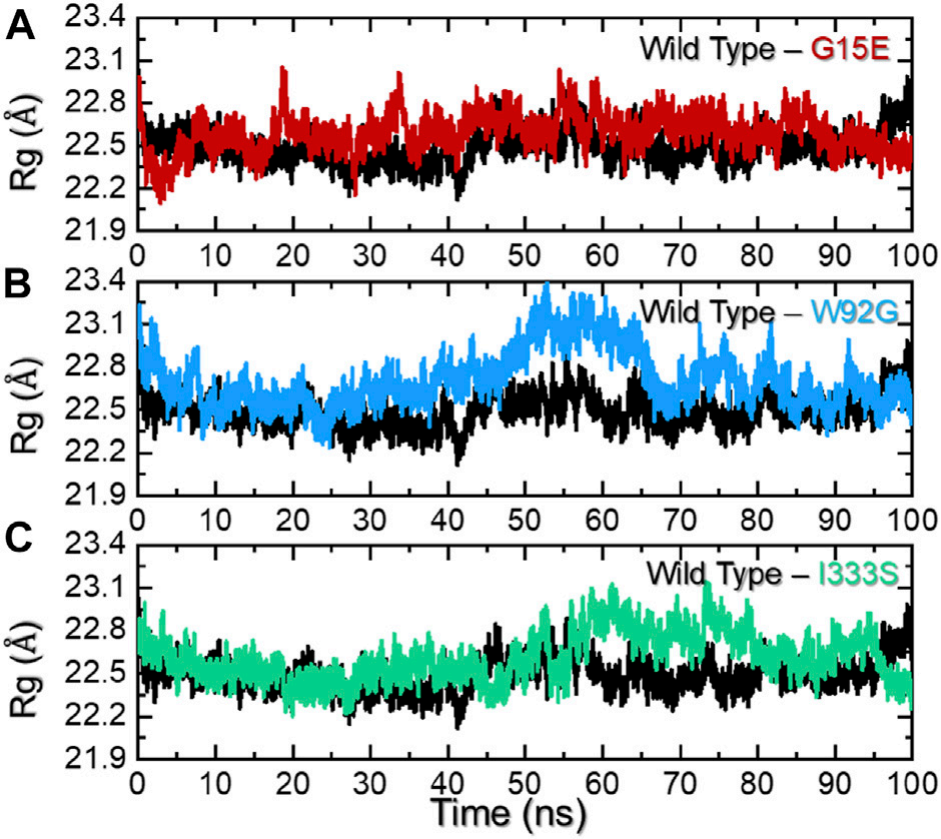
Radius of gyration analysis of the wild-type and mutant complexes**. (A**) Representation of the Rg of the wild type and G15E. **(B)** Representation of the Rg of the wild type and W92G. **(C)** Representation of the Rg of the wild type and I333S. The x-axis represent time in nanoseconds while the y-axis represent Rg in Å.

An assessment of protein packing reveals information regarding the binding and unbinding events that occurred during simulation. These events are steered by the bonding of small molecules with the protein cavity. For instance, this approach has been used previously to see the impact of mutations on the structural compactness when IDH1 binds to its homodimer (Yuan et al., 2017). Herein, to understand the structural compactness, radius of gyration (Rg) was calculated as a function of time. Consistent with the RMSD results, the wild-type complex reported a uniform pattern of Rg during the simulation. There was a slight increase in Rg between 40 and 60 ns, but it then stabilized again until the end of the simulation. The average Rg for this complex was calculated to be 22.5 Å. On the other hand, for the G15E mutant, despite its similar Rg value, , wild-type deviations at different time intervals were observed. This trend can be seen for the whole simulation time period (0–100 ns), which shows maximum unbinding events induced by the mutation. The W82G and I333S mutants demonstrated a similar pattern of Rg for the first 40 ns. With slightly higher Rg values, the two complexes reported patterns similar to those of the wild type. However, W92G experienced a continuous increase in the Rg between 41 and 60 ns and then a decreased back effect was observed until 70 ns. The Rg value for the remaining simulation time remained lower; however, major deviations were reported. On the other hand, I333S reported a gradual increase in the Rg value after 45 ns and continued to follow this pattern until 75 ns. The Rg then again decreased and remained consistent until the end of the simulation. The average Rg values for W92G and I333S were calculated to be 22.80 Å and 22.78 Å, respectively. Interestingly the other reported mutations in the interface site, that is, R132C, R132G, R132H, R132L, and R132S, also demonstrated higher radius of gyration values; thus, further validating our findings in terms of protein compactness (Yuan et al., 2017; Bendahou et al., 2020). The Rg graphs for each complex are shown in Figures 5A–C.

### 3.5 Residues’ flexibility indexing

**FIGURE 6:**
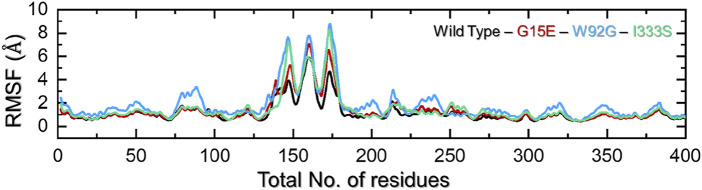
Residues’ flexibility analysis of the wild-type and mutant complexes. The x-axis represents time in total number of residues while the y-axis represent RMSF in Å.

Residues’ flexibility indexing is a key assessment to understand the role of each residue in different biological functions. The flexibility can be applied in different domains, such as molecular recognition, drug binding, cascade signaling, protein coupling, enzyme engineering, and protein designs. To estimate the residual flexibility of each complex, we calculated root mean square fluctuation (RMSF). As shown in Figure 5, all of the complexes demonstrated a more similar pattern of RMSF except for the regions between 125 and175 for all of the complexes. The flexibility at this portion (125–175) revealed a different pattern, which shows the impact of a particular mutation on the protein’s internal dynamics. The wild type in this region demonstrates minimal RMSF, while the three mutations reported maximum RMSF. This consequently shows the altered dynamics and residue flexibility by the induced mutations in the structure, thus altering the binding of small molecules and function. This overall higher flexibility with the loss of compactness and intramolecular hydrogen bonds makes these mutations more deleterious than the other mutations. The findings are prevalidated by the previous literature where increased flexibility was observed mediated by different mutations. Moreover, other diseases mutations are reported to decrease the binding either due to increasing the cavity space or affecting the on/off switch, which consequently increases/decreases the distance between essential atoms (Dehury et al., 2020b; Dehury et al., 2020c). The RMSF of each complex is shown in Figure 6.

### 3.6 Hydrogen bonding analysis

**FIGURE 7:**
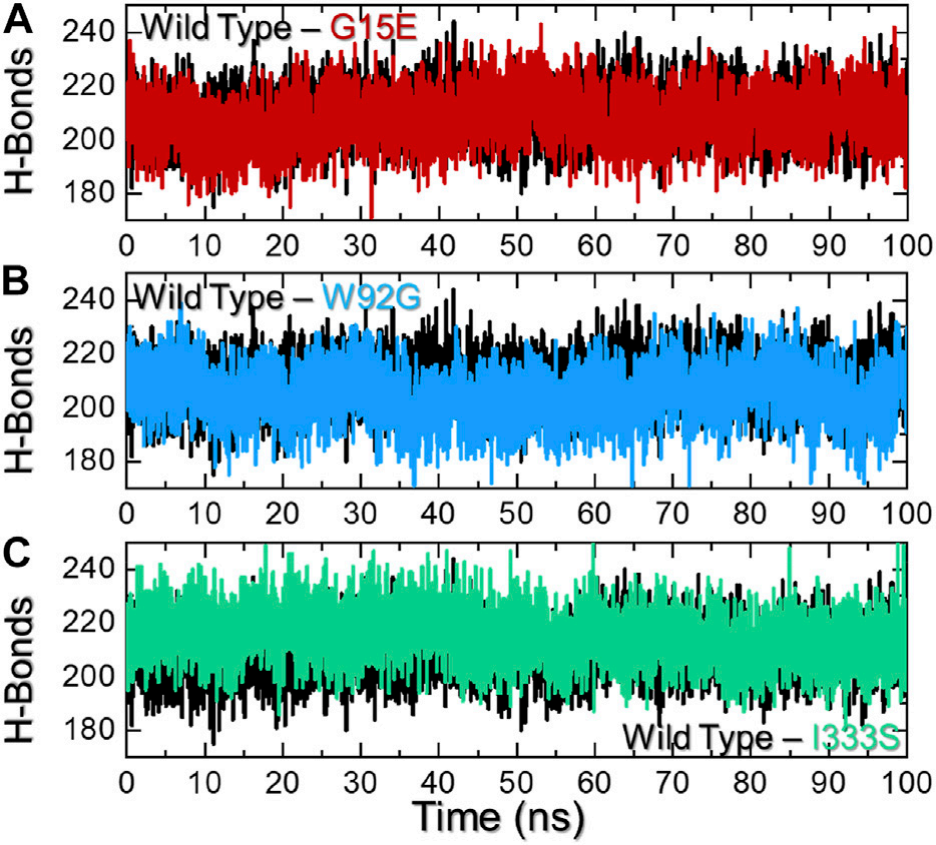
Hydrogen bonding analysis of the wild-type and mutant complexes. **(A**) Representation of H-bonds of the wild type and G15E. **(B)** Representation of H-bonds of the wild type and W92G. **(C)** Representation of H-bonds of the wild type and I333S. The x-axis represents time in nanoseconds, while the y-axis represents H-bond population.

Macromolecular complexes, particularly protein–protein coupling, are primarily driven by numerous factors, among which hydrogen bonding and hydrophobic contacts are essential. The environment of protein interfaces is enriched with water molecules that work with the residues to form hydrogen bonds (Chen et al., 2016). The mechanisms underlying protein–protein interaction as well as the ramifications for hydrogen bonding are unclear (Chodera and Mobley, 2013). Whether hydrogen bonds govern protein–protein docking, in particular, is a long-standing concern, and the mechanism is poorly understood (Patil et al., 2010; Olsson et al., 2011). Therefore, it is important to understand the hydrogen bonding landscape in protein–protein association. For instance, previously, hydrogen bonding was predicted to estimate the strength of the association between two molecules. As shown in Figure 7A, the wild type reported an average of 215 hydrogen bonds, while the G15E complex reported 212 hydrogen bonds over the simulation time. On the other hand, the W92G complex reported 209 average hydrogen bonds in contrast to the wild type. The hydrogen bonds in the wild type and I333S were comparable. In the case of I333S, average hydrogen bonds were calculated to be 215, same as that of the wild type. This demonstrate that these mutations alter the internal dynamics, consequently altering the hydrogen bonding network and causes resistance to the drug. The hydrogen bonding graphs of all of the complexes are shown in Figures 7A–C.

### 3.7 Binding free energy calculation

Binding free energy calculation determines the accurate binding strength and conformation of the small molecule. It is an essential estimation to re-evaluate the docking predictions by considering the highest accuracy and reliability than the conventional docking and alchemical methods. It is a widely used approach to explore the interaction strength and reveal the key binding feature, which steers the overall binding. Considering the applicability of the MM/GBSA approach, we also estimated the total binding energy for the wild-type and mutant complex. As shown in Table 3, the vdW for the wild type was estimated to be −40.78 ± 0.045 kcal/mol, while for the mutant it was −35.13 ± 0.054 kcal/mol reported by G15E mutations, −38.07 ± 0.053 kcal/mol reported by W92G, and −36.46 ± 0.06 kcal/mol calculated for the I333S mutant. This shows the loss of important interacting contacts that remained conserved in the wild type. On the other hand, the electrostatic energy determined an inverted trend. In the case of wild type, the electrostatic energy was calculated to be 3.55 ± 0.034 kcal/mol, while for the mutations −1.23 ± 0.057 kcal/mol (G15E) and −2.53 ± 0.052 kcal/mol (W92G), and −0.99 ± 0.06 kcal/mol for I333S mutant was calculated. This shows that due to these mutations the electrostatic contacts are increased, which may consequently alter the binding. Moreover, ∆G total was reported to be −34.77 ± 0.036 kcal/mol for the wild type and −34.07 ± 0.051 kcal/mol for W92G. The total binding energy of the wild type and G15E is comparable. The two mutants, that is, G15E and I333S, demonstrated a significant decrease in the ∆G total. The ∆G total for the G15E mutant was calculated to be −30.90 ± 0.041 kcal/mol, while for I333S, the ∆G total was estimated to be −31.91 ± 0.04 kcal/mol. This consequently shows the impact of these deleterious substitutions on the binding of the small molecule. The other parameters of the total binding free energy are given in Table 3.

**TABLE 3:** Total binding free energy for the wild-type, G15E, W92G, and I333S complexes. All of the values are calculated in kcal/mol.

Parameters	Wild type	G15E	W92G	I333S
VDWAALS	−40.78 ± 0.045	−35.13 ± 0.054	−38.07 ± 0.053	−36.46 ± 0.06
EEL	3.55 ± 0.034	−1.23 ± 0.057	−2.53 ± 0.052	−0.99 ± 0.06
EGB	7.22 ± 0.023	9.64 ± 0.040	10.55 ± 0.056	9.73 ± 0.04
ESURF	−4.77 ± 0.003	−4.18 ± 0.007	−4.02 ± 0.005	−4.19 ± 0.008
Delta G Gas	−37.23 ± 0.037	−36.36 ± 0.046	−40.61 ± 0.071	−37.45 ± 0.005
Delta G Solv	2.45 ± 0.023	5.46 ± 0.043	6.53 ± 0.056	5.53 ± 0.005
Delta Total	−34.77 ± 0.036	−30.90 ± 0.041	−34.07 ± 0.051	−31.91 ± 0.04

### 3.8 Clustering of protein’s motion

**FIGURE 8:**
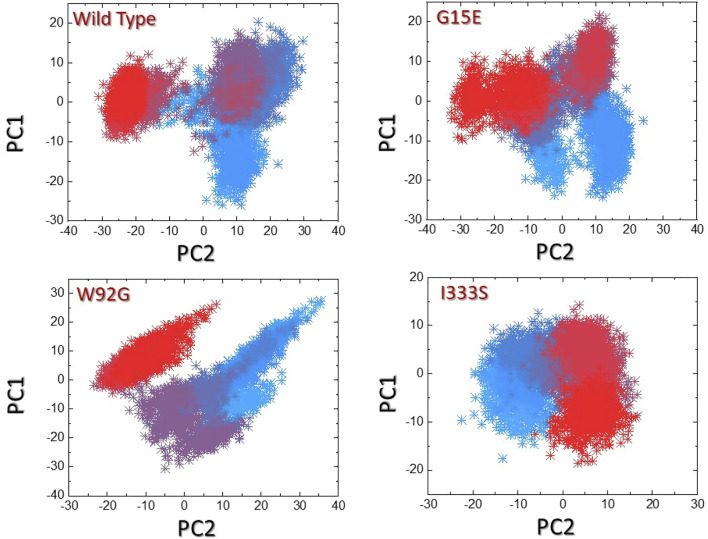
Principal component analysis of the wild-type and mutant complexes. **(A**) Representation of PCA of the wild type. **(B)** Representation of PCA of G15E. **(C)** Representation of PCA of W92G. **(D)** Representation of PCA of I333S. The red to sky blue represent the different conformation states, whereas the transition states are represented by dark purple.

We used the principal component analysis (PCA) to cluster the protein motions in the simulation trajectories. The two PCs, that is, PC1 and PC2, reflected these motions in two dimensions. The distributed principal components for each complex are given in Figures 8A–D. The first three eigenvectors contributed 54% of the total motion, while the rest was contributed by the other eigenvectors. In contrast, the three mutants, that is, G15E the first three eigenvectors contributed 49%, W92G reported 48%, and I333S reported 44% of the total motion by the first three eigenvectors. The rest demonstrated localized motion by these complexes. The conformational transition can be easily separated from each other by sky blue and red colors. This shows that these mutations had caused different arrangements to bind small molecules compared to the wild type, where the total internal motion is decreased, thus consequently producing minimal binding effects.

### 3.9 Free energy landscape analysis

**FIGURE 9:**
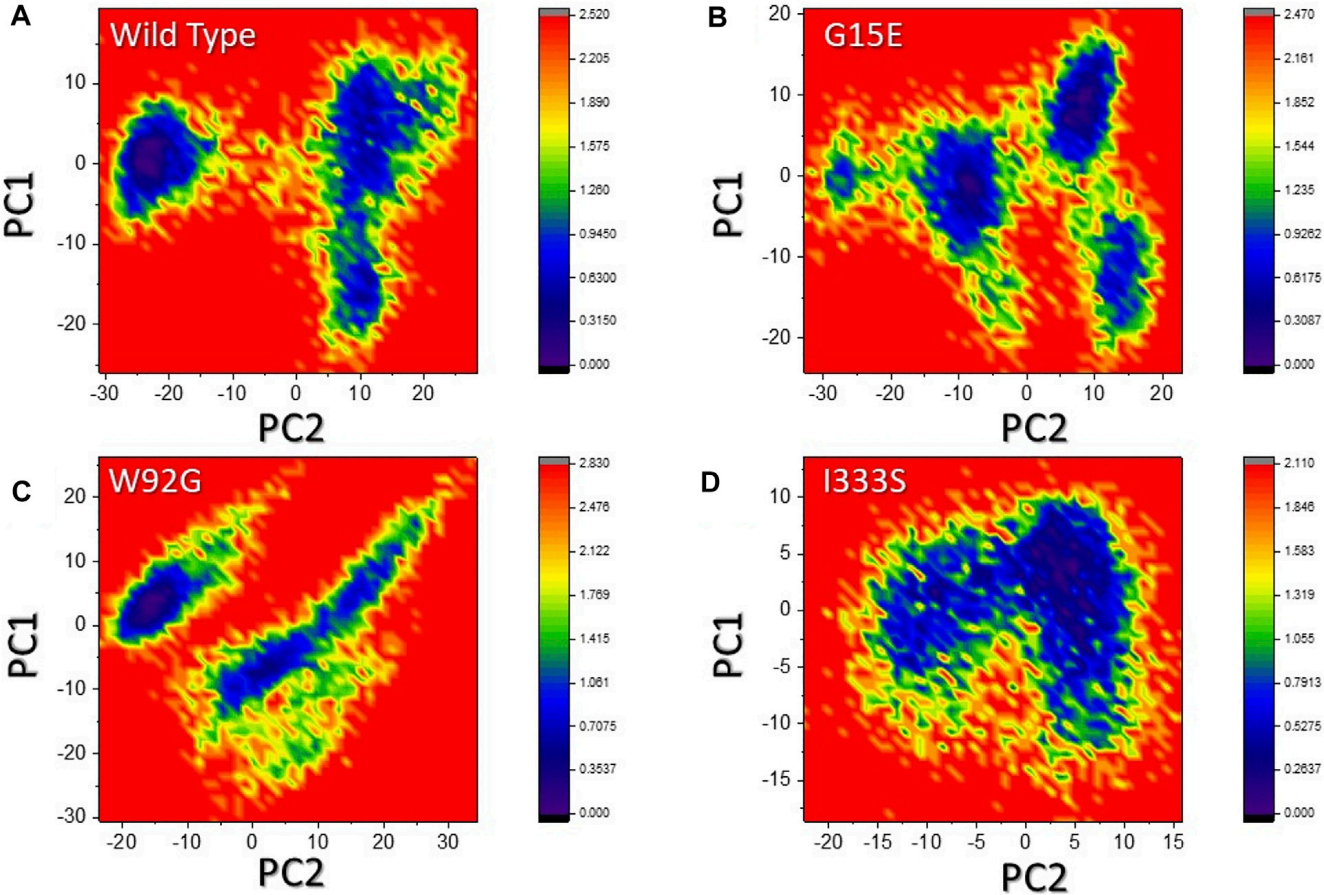
Free energy landscape analysis of the wild-type and mutant complexes. **(A**) Representation of DEL of the wild type. **(B)** Representation of FEL of G15E. **(C)** Representation of the FEL of W92G. **(D)** Representation of the FEL of I333S. The dark regions in each show the lowest-energy conformation where the conformational states are separated by subspace in each complex.

The two PCs were then mapped to identify the stable and metastable states for each complex. As shown in Figures 9A–D, the wild type attained one lowest-energy conformation, while G15E and W92G attained two lowest-energy conformations. I333S also attained a single lowest-energy state. The conformational transition in each complex is separated by a subspace. This shows that the mutations had caused a different arrangement to bind small molecules compared to the wild type, thus consequently producing minimal binding effects.

## 4 Conclusion

The current study used genomic mutation screening and molecular simulation methods to identify the most detrimental mutations in the IDH1 gene. The investigation of a large number of mutations revealed that three mutations, G15E, W92G, and I333S, are the most deleterious and highly destabilizing, which can affect the binding of a drug. These mutations primarily affect the binding of the drug with IDH, thus consequently reducing the efficacy of the already approved drug. Further validations such as molecular docking and dynamics simulation demonstrated that these mutations do not only affect the stability but also alter the bonding network. In addition, the BFE was also observed to have been reduced due to conformational changes mediated by these mutations. In sum, the current mutations contribute to drug resistance in glioma, and the atomic features explored in this study could be used for structure-based drug designing against resistant glioma.

## Data Availability

The original contributions presented in the study are included in the article/Supplementary Material. Further inquiries can be directed to the corresponding authors.

## Abbreviations

PredictSNP, predict single nucleotide polymorphism; Polyphen 2, Polymorphism Phenotype version 2; SNAP, Screening of nonacceptable polymorphism; PANTHER, Protein Analysis Through Evolutionary Relationship; MAPP, Multivariate Analysis of Protein Polymorphism; PhD-SNP, predictor of Human Deleterious Single Nucleotide Polymorphism; IDH, isocitrate dehydrogenase (IDH) encoding genes; PCA, principal component analysis; FEL, free energy landscape; RMSD, root mean square deviation; RMSF, root mean square fluctuation; and Rg, radius of gyration.
