# Supplementary material for: Sequence-structure functional implications and molecular simulation of high deleterious nonsynonymous substitutions in IDH1 revealed the mechanism of drug resistance in glioma
Source: Front Pharmacol. 2022 Sep 16;13:927570. doi: 10.3389/fphar.2022.927570 (PMC9523485; doi:10.3389/fphar.2022.927570)
Supplement: Supplementary file 1 [file DataSheet1.docx]

**Supplementary information**

**Sequence-Structure functional Implications and molecular simulation of high deleterious Non-Synonymous substitutions revealed the mechanism of drug resistance in glioma**

Muhammad Suleman^1#^, Syeda Umme-i-Hani^2^, Muhammad Salman^3^, Mohammed Aljuaid^4^, Abbas Khan^5#^, Arshad Iqbal^1^, Zahid Hussain^1^, Syed Shujait Ali^1^, Yasir Waheed^6*^, Dong-Qing Wei^5,7, 8*^

^1^Centre for Biotechnology and Microbiology, University of Swat, Khyber Pakhtunkhwa, Pakistan.

^2^Punjab Medical College, Faisalabad, Punjab, Pakistan. [Tamimrai18@gmail.com](mailto:Tamimrai18@gmail.com).

^3^Rashid Latif Medical College, Lahore, Punjab, Pakistan.

^4^Department of Health Administration, College of Business Administration, King Saud University. [maljuaid@ksu.edu.sa](mailto:maljuaid@ksu.edu.sa).

^5^Department of Bioinformatics and Biological Statistics, School of Life Sciences and Biotechnology, Shanghai Jiao Tong University, Shanghai, 200240, P.R. China.

^6^Foundation University Medical College, Foundation University Islamabad, Islamabad, Pakistan. [yasir_waheed_199@hotmail.com](mailto:yasir_waheed_199@hotmail.com)

^7^State Key Laboratory of Microbial Metabolism, Shanghai-Islamabad-Belgrade Joint Innovation Center on Antibacterial Resistances, Joint Laboratory of International Cooperation in Metabolic and Developmental Sciences, Ministry of Education and School of Life Sciences and Biotechnology, Shanghai Jiao Tong University, Shanghai 200030, P.R. China

^8^Peng Cheng Laboratory, Vanke Cloud City Phase I Building 8, Xili Street, Nashan District, Shenzhen, Guangdong, 518055, P.R China.

***Corresponding authors**

[yasir_waheed_199@hotmail.com](mailto:yasir_waheed_199@hotmail.com) / [dqwei@sjtu.edu.cn](mailto:dqwei@sjtu.edu.cn)

**Table 1. Analysis of nSNPS using different servers.**

| **Wild residue** | **Position** | **Target residue** | **PredictSNP prediction** | **PredictSNP expected accuracy** | **MAPP prediction** | **MAPP expected accuracye** |
| --- | --- | --- | --- | --- | --- | --- |
| S | 2 | P | NEUTRAL | 0.61421911 | DELETERIOUS | 0.48350825 |
| K | 4 | Q | NEUTRAL | 0.6025641 | NEUTRAL | 0.6778169 |
| S | 6 | G | NEUTRAL | 0.73688811 | DELETERIOUS | 0.55997001 |
| G | 8 | S | NEUTRAL | 0.68365861 | NEUTRAL | 0.79030837 |
| E | 12 | Q | DELETERIOUS | 0.7556615 | NEUTRAL | 0.64229075 |
| Q | 14 | R | NEUTRAL | 0.6025641 | DELETERIOUS | 0.42653673 |
| G | 15 | E | DELETERIOUS | 0.86908365 | DELETERIOUS | 0.87706147 |
| D | 16 | H | DELETERIOUS | 0.86908365 | DELETERIOUS | 0.71814093 |
| T | 19 | A | DELETERIOUS | 0.50595948 | DELETERIOUS | 0.62143928 |
| R | 20 | Q | DELETERIOUS | 0.60548272 | NEUTRAL | 0.64229075 |
| R | 20 | L | DELETERIOUS | 0.86908365 | DELETERIOUS | 0.55997001 |
| I | 22 | V | NEUTRAL | 0.75291375 | NEUTRAL | 0.65022026 |
| W | 23 | C | DELETERIOUS | 0.86908365 | DELETERIOUS | 0.65667166 |
| K | 27 | Q | DELETERIOUS | 0.50595948 | NEUTRAL | 0.65903084 |
| L | 30 | V | DELETERIOUS | 0.50595948 | DELETERIOUS | 0.40929535 |
| L | 30 | I | NEUTRAL | 0.6025641 | NEUTRAL | 0.74823944 |
| I | 31 | V | NEUTRAL | 0.63151762 | DELETERIOUS | 0.5089955 |
| F | 32 | V | NEUTRAL | 0.65307311 | NEUTRAL | 0.64229075 |
| P | 33 | S | DELETERIOUS | 0.60548272 | DELETERIOUS | 0.58770615 |
| Y | 34 | C | DELETERIOUS | 0.86908365 | DELETERIOUS | 0.42653673 |
| V | 35 | A | DELETERIOUS | 0.86908365 | DELETERIOUS | 0.62143928 |
| V | 35 | M | DELETERIOUS | 0.54946365 | DELETERIOUS | 0.65667166 |
| D | 38 | N | NEUTRAL | 0.73688811 | NEUTRAL | 0.78325991 |
| S | 41 | G | DELETERIOUS | 0.50595948 | DELETERIOUS | 0.62143928 |
| Y | 42 | C | DELETERIOUS | 0.86908365 | DELETERIOUS | 0.65667166 |
| D | 43 | A | DELETERIOUS | 0.86908365 | DELETERIOUS | 0.78335832 |
| R | 49 | H | DELETERIOUS | 0.86908365 | DELETERIOUS | 0.65667166 |
| R | 49 | C | DELETERIOUS | 0.86908365 | DELETERIOUS | 0.85682159 |
| R | 49 | P | DELETERIOUS | 0.86908365 | DELETERIOUS | 0.9197901 |
| A | 51 | G | NEUTRAL | 0.6025641 | NEUTRAL | 0.64229075 |
| D | 54 | N | DELETERIOUS | 0.7556615 | DELETERIOUS | 0.63268366 |
| Q | 55 | P | DELETERIOUS | 0.7556615 | DELETERIOUS | 0.78335832 |
| A | 61 | T | DELETERIOUS | 0.50595948 | DELETERIOUS | 0.76611694 |
| E | 62 | A | NEUTRAL | 0.73834499 | DELETERIOUS | 0.42653673 |
| E | 62 | K | NEUTRAL | 0.75203963 | NEUTRAL | 0.76123348 |
| H | 67 | R | DELETERIOUS | 0.71871275 | DELETERIOUS | 0.63268366 |
| H | 67 | Q | DELETERIOUS | 0.60548272 | NEUTRAL | 0.70484581 |
| N | 68 | D | NEUTRAL | 0.6025641 | DELETERIOUS | 0.40929535 |
| G | 70 | S | DELETERIOUS | 0.60548272 | NEUTRAL | 0.77004405 |
| V | 71 | I | NEUTRAL | 0.82622462 | NEUTRAL | 0.76563877 |
| I | 76 | V | NEUTRAL | 0.6025641 | DELETERIOUS | 0.48350825 |
| I | 76 | T | DELETERIOUS | 0.86908365 | DELETERIOUS | 0.58770615 |
| R | 82 | S | DELETERIOUS | 0.86908365 | DELETERIOUS | 0.76536732 |
| R | 82 | K | DELETERIOUS | 0.7556615 | NEUTRAL | 0.63348018 |
| R | 82 | M | DELETERIOUS | 0.86908365 | DELETERIOUS | 0.76536732 |
| V | 83 | F | DELETERIOUS | 0.86908365 | DELETERIOUS | 0.62143928 |
| E | 84 | D | NEUTRAL | 0.6025641 | DELETERIOUS | 0.58889722 |
| E | 85 | G | DELETERIOUS | 0.86908365 | DELETERIOUS | 0.78335832 |
| F | 86 | L | DELETERIOUS | 0.7556615 | DELETERIOUS | 0.58889722 |
| L | 88 | F | DELETERIOUS | 0.86908365 | DELETERIOUS | 0.65667166 |
| M | 91 | I | DELETERIOUS | 0.71871275 | DELETERIOUS | 0.48350825 |
| M | 91 | R | DELETERIOUS | 0.86908365 | DELETERIOUS | 0.81934033 |
| M | 91 | L | DELETERIOUS | 0.50595948 | NEUTRAL | 0.70484581 |
| M | 91 | T | DELETERIOUS | 0.86908365 | DELETERIOUS | 0.57121439 |
| W | 92 | G | DELETERIOUS | 0.86908365 | DELETERIOUS | 0.84182909 |
| W | 92 | R | DELETERIOUS | 0.86908365 | DELETERIOUS | 0.87706147 |
| K | 93 | E | DELETERIOUS | 0.50595948 | NEUTRAL | 0.64229075 |
| N | 96 | H | DELETERIOUS | 0.86908365 | DELETERIOUS | 0.57121439 |
| T | 98 | N | DELETERIOUS | 0.86908365 | DELETERIOUS | 0.40929535 |
| I | 99 | M | DELETERIOUS | 0.71871275 | DELETERIOUS | 0.62143928 |
| N | 101 | Y | DELETERIOUS | 0.86908365 | DELETERIOUS | 0.57314329 |
| T | 106 | M | DELETERIOUS | 0.86908365 | DELETERIOUS | 0.76536732 |
| V | 107 | I | NEUTRAL | 0.82622462 | NEUTRAL | 0.63348018 |
| F | 108 | V | DELETERIOUS | 0.86908365 | DELETERIOUS | 0.58889722 |
| R | 109 | K | DELETERIOUS | 0.86908365 | DELETERIOUS | 0.55997001 |
| I | 112 | V | NEUTRAL | 0.6025641 | DELETERIOUS | 0.62143928 |
| I | 113 | S | DELETERIOUS | 0.86908365 | DELETERIOUS | 0.5089955 |
| I | 113 | T | DELETERIOUS | 0.7556615 | DELETERIOUS | 0.46176912 |
| I | 117 | V | NEUTRAL | 0.82622462 | NEUTRAL | 0.64229075 |
| R | 119 | W | DELETERIOUS | 0.86908365 | DELETERIOUS | 0.40929535 |
| R | 119 | Q | DELETERIOUS | 0.71871275 | NEUTRAL | 0.73127753 |
| S | 122 | N | NEUTRAL | 0.73688811 | NEUTRAL | 0.74273128 |
| G | 123 | R | DELETERIOUS | 0.60548272 | DELETERIOUS | 0.57314329 |
| V | 125 | L | NEUTRAL | 0.65307311 | NEUTRAL | 0.65903084 |
| P | 127 | T | DELETERIOUS | 0.7556615 | DELETERIOUS | 0.58770615 |
| I | 130 | V | NEUTRAL | 0.73834499 | DELETERIOUS | 0.42653673 |
| I | 130 | T | DELETERIOUS | 0.71871275 | DELETERIOUS | 0.76086957 |
| D | 137 | N | DELETERIOUS | 0.7556615 | NEUTRAL | 0.65022026 |
| T | 142 | I | NEUTRAL | 0.62831343 | NEUTRAL | 0.70484581 |
| P | 147 | S | NEUTRAL | 0.75203963 | NEUTRAL | 0.65903084 |
| P | 149 | L | DELETERIOUS | 0.71871275 | DELETERIOUS | 0.42653673 |
| P | 149 | T | DELETERIOUS | 0.50595948 | DELETERIOUS | 0.40929535 |
| G | 150 | R | DELETERIOUS | 0.86908365 | DELETERIOUS | 0.85682159 |
| V | 152 | G | DELETERIOUS | 0.7556615 | DELETERIOUS | 0.58770615 |
| E | 153 | Q | NEUTRAL | 0.82622462 | NEUTRAL | 0.79030837 |
| I | 154 | R | DELETERIOUS | 0.86908365 | DELETERIOUS | 0.87706147 |
| P | 158 | S | NEUTRAL | 0.82622462 | NEUTRAL | 0.65022026 |
| D | 160 | Y | DELETERIOUS | 0.86908365 | DELETERIOUS | 0.46176912 |
| G | 161 | R | DELETERIOUS | 0.7556615 | DELETERIOUS | 0.62143928 |
| T | 162 | S | NEUTRAL | 0.82622462 | NEUTRAL | 0.85110132 |
| T | 166 | I | NEUTRAL | 0.82622462 | NEUTRAL | 0.70484581 |
| E | 174 | D | NEUTRAL | 0.82622462 | NEUTRAL | 0.70484581 |
| G | 175 | D | NEUTRAL | 0.71278765 |  |  |
| G | 177 | D | DELETERIOUS | 0.86908365 | DELETERIOUS | 0.85682159 |
| V | 178 | I | NEUTRAL | 0.82622462 | NEUTRAL | 0.71013216 |
| M | 180 | V | NEUTRAL | 0.6025641 | DELETERIOUS | 0.5089955 |
| M | 180 | L | NEUTRAL | 0.82622462 | NEUTRAL | 0.78325991 |
| G | 181 | V | DELETERIOUS | 0.60548272 | NEUTRAL | 0.65022026 |
| M | 182 | I | DELETERIOUS | 0.60548272 | NEUTRAL | 0.64229075 |
| Y | 183 | H | NEUTRAL | 0.62529138 | NEUTRAL | 0.73127753 |
| Y | 183 | C | DELETERIOUS | 0.86908365 | DELETERIOUS | 0.48350825 |
| I | 189 | V | NEUTRAL | 0.65307311 | DELETERIOUS | 0.5089955 |
| E | 190 | K | NEUTRAL | 0.75203963 | NEUTRAL | 0.78854626 |
| A | 193 | T | DELETERIOUS | 0.86908365 | DELETERIOUS | 0.85832084 |
| H | 194 | Y | NEUTRAL | 0.63151762 | DELETERIOUS | 0.58889722 |
| Q | 198 | R | NEUTRAL | 0.75203963 | NEUTRAL | 0.6778169 |
| A | 200 | S | NEUTRAL | 0.62831343 | DELETERIOUS | 0.55997001 |
| L | 201 | V | DELETERIOUS | 0.50595948 | DELETERIOUS | 0.57121439 |
| S | 202 | A | NEUTRAL | 0.82622462 | NEUTRAL | 0.65903084 |
| K | 203 | R | NEUTRAL | 0.82622462 | NEUTRAL | 0.79559471 |
| G | 204 | V | DELETERIOUS | 0.50595948 | DELETERIOUS | 0.63268366 |
| L | 207 | W | DELETERIOUS | 0.86908365 | DELETERIOUS | 0.55997001 |
| Y | 208 | H | DELETERIOUS | 0.86908365 | DELETERIOUS | 0.5089955 |
| Y | 208 | C | DELETERIOUS | 0.86908365 | DELETERIOUS | 0.57121439 |
| T | 214 | S | DELETERIOUS | 0.86908365 | DELETERIOUS | 0.75112444 |
| K | 217 | R | DELETERIOUS | 0.60697259 | NEUTRAL | 0.70484581 |
| Y | 219 | H | DELETERIOUS | 0.86908365 | DELETERIOUS | 0.75112444 |
| Y | 219 | C | DELETERIOUS | 0.86908365 | DELETERIOUS | 0.75112444 |
| D | 220 | G | DELETERIOUS | 0.86908365 | DELETERIOUS | 0.71814093 |
| R | 222 | H | DELETERIOUS | 0.60548272 | NEUTRAL | 0.78325991 |
| R | 222 | C | DELETERIOUS | 0.65494636 | NEUTRAL | 0.64229075 |
| E | 229 | V | DELETERIOUS | 0.7556615 | DELETERIOUS | 0.65667166 |
| Y | 231 | H | DELETERIOUS | 0.86908365 | DELETERIOUS | 0.8065967 |
| D | 232 | N | NEUTRAL | 0.73688811 | NEUTRAL | 0.76123348 |
| K | 233 | M | NEUTRAL | 0.73834499 | NEUTRAL | 0.74977974 |
| Y | 235 | C | DELETERIOUS | 0.86908365 | DELETERIOUS | 0.71814093 |
| K | 236 | Q | NEUTRAL | 0.63151762 | NEUTRAL | 0.6778169 |
| Q | 238 | H | NEUTRAL | 0.65307311 | NEUTRAL | 0.73127753 |
| F | 239 | L | DELETERIOUS | 0.86908365 | DELETERIOUS | 0.63268366 |
| Q | 242 | H | NEUTRAL | 0.82622462 | NEUTRAL | 0.70484581 |
| K | 243 | E | NEUTRAL | 0.6025641 | DELETERIOUS | 0.5089955 |
| K | 243 | R | DELETERIOUS | 0.60548272 | DELETERIOUS | 0.5089955 |
| Y | 246 | H | DELETERIOUS | 0.86908365 | DELETERIOUS | 0.78335832 |
| D | 252 | N | DELETERIOUS | 0.7556615 | DELETERIOUS | 0.76536732 |
| A | 256 | V | DELETERIOUS | 0.86908365 | DELETERIOUS | 0.62143928 |
| A | 256 | S | DELETERIOUS | 0.7556615 | DELETERIOUS | 0.62143928 |
| K | 260 | N | DELETERIOUS | 0.86908365 | DELETERIOUS | 0.63268366 |
| G | 263 | E | DELETERIOUS | 0.86908365 | DELETERIOUS | 0.87706147 |
| G | 264 | A | DELETERIOUS | 0.50595948 | DELETERIOUS | 0.65667166 |
| I | 266 | V | NEUTRAL | 0.82622462 | NEUTRAL | 0.85110132 |
| D | 273 | G | DELETERIOUS | 0.86908365 | DELETERIOUS | 0.77494374 |
| G | 274 | S | DELETERIOUS | 0.86908365 | DELETERIOUS | 0.57121439 |
| V | 276 | M | DELETERIOUS | 0.78903456 | DELETERIOUS | 0.76536732 |
| S | 278 | P | DELETERIOUS | 0.86908365 | DELETERIOUS | 0.7711928 |
| S | 278 | L | DELETERIOUS | 0.86908365 | DELETERIOUS | 0.9137931 |
| D | 279 | H | DELETERIOUS | 0.86908365 | DELETERIOUS | 0.71814093 |
| S | 280 | C | NEUTRAL | 0.75291375 | NEUTRAL | 0.63348018 |
| S | 280 | F | NEUTRAL | 0.73688811 | NEUTRAL | 0.74185022 |
| M | 291 | T | DELETERIOUS | 0.86908365 | DELETERIOUS | 0.57121439 |
| T | 292 | I | DELETERIOUS | 0.86908365 | DELETERIOUS | 0.58770615 |
| S | 293 | I | DELETERIOUS | 0.86908365 | DELETERIOUS | 0.78335832 |
| V | 294 | M | DELETERIOUS | 0.60548272 | NEUTRAL | 0.65286344 |
| P | 298 | L | DELETERIOUS | 0.86908365 | DELETERIOUS | 0.57314329 |
| G | 300 | V | DELETERIOUS | 0.86908365 | DELETERIOUS | 0.8065967 |
| G | 300 | D | DELETERIOUS | 0.7556615 | DELETERIOUS | 0.57314329 |
| T | 302 | A | DELETERIOUS | 0.50595948 | DELETERIOUS | 0.55997001 |
| E | 304 | G | DELETERIOUS | 0.71871275 | DELETERIOUS | 0.63268366 |
| A | 305 | T | NEUTRAL | 0.63151762 | NEUTRAL | 0.71013216 |
| E | 306 | A | DELETERIOUS | 0.86908365 | DELETERIOUS | 0.76536732 |
| H | 309 | R | DELETERIOUS | 0.86908365 | DELETERIOUS | 0.8065967 |
| H | 309 | Q | DELETERIOUS | 0.86908365 | DELETERIOUS | 0.57121439 |
| G | 310 | R | DELETERIOUS | 0.86908365 | DELETERIOUS | 0.87706147 |
| R | 314 | H | DELETERIOUS | 0.7556615 | NEUTRAL | 0.64229075 |
| R | 314 | C | DELETERIOUS | 0.86908365 | DELETERIOUS | 0.63268366 |
| H | 315 | D | DELETERIOUS | 0.86908365 | DELETERIOUS | 0.76536732 |
| R | 317 | H | DELETERIOUS | 0.65494636 | NEUTRAL | 0.74823944 |
| R | 317 | C | DELETERIOUS | 0.86908365 | DELETERIOUS | 0.48350825 |
| R | 317 | L | DELETERIOUS | 0.86908365 | DELETERIOUS | 0.40929535 |
| M | 318 | T | NEUTRAL | 0.65307311 | DELETERIOUS | 0.42653673 |
| K | 321 | E | DELETERIOUS | 0.54946365 | DELETERIOUS | 0.42653673 |
| T | 325 | M | DELETERIOUS | 0.86908365 | DELETERIOUS | 0.75112444 |
| S | 326 | P | DELETERIOUS | 0.7556615 | DELETERIOUS | 0.9137931 |
| T | 327 | I | DELETERIOUS | 0.7556615 | DELETERIOUS | 0.58770615 |
| N | 328 | S | DELETERIOUS | 0.86908365 | DELETERIOUS | 0.63268366 |
| N | 328 | K | DELETERIOUS | 0.86908365 | DELETERIOUS | 0.81934033 |
| I | 330 | T | DELETERIOUS | 0.86908365 | DELETERIOUS | 0.48350825 |
| I | 333 | S | DELETERIOUS | 0.86908365 | DELETERIOUS | 0.76536732 |
| A | 335 | S | DELETERIOUS | 0.60548272 | DELETERIOUS | 0.57121439 |
| R | 338 | S | NEUTRAL | 0.6025641 | NEUTRAL | 0.74273128 |
| G | 339 | R | DELETERIOUS | 0.86908365 | DELETERIOUS | 0.87706147 |
| A | 341 | V | NEUTRAL | 0.75203963 | NEUTRAL | 0.64229075 |
| A | 341 | P | DELETERIOUS | 0.60697259 | DELETERIOUS | 0.62143928 |
| A | 341 | D | NEUTRAL | 0.74796037 | NEUTRAL | 0.64229075 |
| L | 346 | P | DELETERIOUS | 0.86908365 | DELETERIOUS | 0.75112444 |
| E | 351 | G | NEUTRAL | 0.65307311 | NEUTRAL | 0.65286344 |
| E | 351 | K | NEUTRAL | 0.82622462 | NEUTRAL | 0.7596831 |
| A | 353 | G | NEUTRAL | 0.82622462 | NEUTRAL | 0.64229075 |
| A | 353 | D | NEUTRAL | 0.73688811 | NEUTRAL | 0.6778169 |
| F | 354 | V | NEUTRAL | 0.73834499 | DELETERIOUS | 0.5089955 |
| F | 354 | L | NEUTRAL | 0.82622462 | NEUTRAL | 0.73480176 |
| A | 356 | V | NEUTRAL | 0.74796037 | NEUTRAL | 0.64229075 |
| S | 363 | A | NEUTRAL | 0.63151762 | DELETERIOUS | 0.57314329 |
| I | 364 | V | NEUTRAL | 0.82622462 | NEUTRAL | 0.71742958 |
| I | 364 | L | NEUTRAL | 0.63151762 | NEUTRAL | 0.63348018 |
| E | 365 | V | DELETERIOUS | 0.60548272 | DELETERIOUS | 0.57314329 |
| I | 367 | V | NEUTRAL | 0.82622462 | NEUTRAL | 0.72246696 |
| I | 367 | T | DELETERIOUS | 0.86908365 | DELETERIOUS | 0.62143928 |
| A | 369 | V | NEUTRAL | 0.6025641 | DELETERIOUS | 0.48350825 |
| G | 370 | V | DELETERIOUS | 0.86908365 | DELETERIOUS | 0.84182909 |
| M | 372 | T | DELETERIOUS | 0.86908365 | DELETERIOUS | 0.57314329 |
| T | 373 | I | DELETERIOUS | 0.86908365 | DELETERIOUS | 0.9137931 |
| T | 373 | N | DELETERIOUS | 0.86908365 | DELETERIOUS | 0.76086957 |
| K | 374 | E | DELETERIOUS | 0.71871275 | DELETERIOUS | 0.46176912 |
| A | 378 | V | NEUTRAL | 0.82622462 | NEUTRAL | 0.76563877 |
| A | 378 | G | NEUTRAL | 0.82622462 | NEUTRAL | 0.74977974 |
| L | 383 | F | NEUTRAL | 0.82622462 | NEUTRAL | 0.74977974 |
| P | 384 | L | NEUTRAL | 0.73834499 | NEUTRAL | 0.65022026 |
| N | 385 | S | NEUTRAL | 0.75203963 | NEUTRAL | 0.73480176 |
| N | 385 | D | NEUTRAL | 0.82622462 | NEUTRAL | 0.73480176 |
| R | 388 | H | DELETERIOUS | 0.54946365 |  |  |
| R | 388 | C | DELETERIOUS | 0.7556615 |  |  |
| M | 398 | I | NEUTRAL | 0.82622462 | NEUTRAL | 0.74823944 |
| D | 399 | H | DELETERIOUS | 0.60548272 | DELETERIOUS | 0.40929535 |
| D | 399 | G | DELETERIOUS | 0.52145411 | DELETERIOUS | 0.40929535 |
| L | 401 | P | DELETERIOUS | 0.86908365 | DELETERIOUS | 0.76536732 |
| G | 402 | E | NEUTRAL | 0.82622462 | NEUTRAL | 0.71742958 |
| L | 405 | V | NEUTRAL | 0.6025641 | DELETERIOUS | 0.55997001 |
| K | 408 | Q | NEUTRAL | 0.82622462 | NEUTRAL | 0.78325991 |

**Table 2. Analysis of nSNPS using different servers.**

| **Wild residue** | **Position** | **Target residue** | **PhD-SNP prediction** | **PhD-SNP expected accuracy** | **PolyPhen-1 prediction** | **PolyPhen-1 expected accuracy** |
| --- | --- | --- | --- | --- | --- | --- |
| S | 2 | P | DELETERIOUS | 0.58885542 | NEUTRAL | 0.66884082 |
| K | 4 | Q | NEUTRAL | 0.50824588 | DELETERIOUS | 0.74491225 |
| S | 6 | G | NEUTRAL | 0.71871412 | NEUTRAL | 0.66884082 |
| G | 8 | S | NEUTRAL | 0.58230958 | NEUTRAL | 0.66884082 |
| E | 12 | Q | DELETERIOUS | 0.81731169 | DELETERIOUS | 0.74491225 |
| Q | 14 | R | DELETERIOUS | 0.81731169 | NEUTRAL | 0.66884082 |
| G | 15 | E | DELETERIOUS | 0.81731169 | DELETERIOUS | 0.74491225 |
| D | 16 | H | DELETERIOUS | 0.81731169 | DELETERIOUS | 0.74491225 |
| T | 19 | A | DELETERIOUS | 0.87523992 | NEUTRAL | 0.66884082 |
| R | 20 | Q | DELETERIOUS | 0.81731169 | DELETERIOUS | 0.59445019 |
| R | 20 | L | DELETERIOUS | 0.85822785 | DELETERIOUS | 0.74491225 |
| I | 22 | V | NEUTRAL | 0.55202703 | NEUTRAL | 0.66884082 |
| W | 23 | C | DELETERIOUS | 0.85822785 | DELETERIOUS | 0.74491225 |
| K | 27 | Q | DELETERIOUS | 0.7733853 | DELETERIOUS | 0.59445019 |
| L | 30 | V | DELETERIOUS | 0.85822785 | NEUTRAL | 0.66884082 |
| L | 30 | I | NEUTRAL | 0.50824588 | DELETERIOUS | 0.74491225 |
| I | 31 | V | NEUTRAL | 0.55202703 | NEUTRAL | 0.66884082 |
| F | 32 | V | DELETERIOUS | 0.58885542 | NEUTRAL | 0.66884082 |
| P | 33 | S | DELETERIOUS | 0.73260309 | NEUTRAL | 0.66884082 |
| Y | 34 | C | DELETERIOUS | 0.73260309 | DELETERIOUS | 0.74491225 |
| V | 35 | A | DELETERIOUS | 0.67620995 | DELETERIOUS | 0.59445019 |
| V | 35 | M | DELETERIOUS | 0.67620995 | NEUTRAL | 0.66884082 |
| D | 38 | N | DELETERIOUS | 0.60798122 | NEUTRAL | 0.66884082 |
| S | 41 | G | DELETERIOUS | 0.67620995 | NEUTRAL | 0.66884082 |
| Y | 42 | C | DELETERIOUS | 0.85822785 | DELETERIOUS | 0.74491225 |
| D | 43 | A | DELETERIOUS | 0.88474971 | DELETERIOUS | 0.74491225 |
| R | 49 | H | DELETERIOUS | 0.87523992 | DELETERIOUS | 0.74491225 |
| R | 49 | C | DELETERIOUS | 0.73260309 | DELETERIOUS | 0.74491225 |
| R | 49 | P | DELETERIOUS | 0.73260309 | DELETERIOUS | 0.59445019 |
| A | 51 | G | DELETERIOUS | 0.73260309 | DELETERIOUS | 0.59445019 |
| D | 54 | N | DELETERIOUS | 0.85822785 | DELETERIOUS | 0.74491225 |
| Q | 55 | P | DELETERIOUS | 0.81731169 | DELETERIOUS | 0.74491225 |
| A | 61 | T | DELETERIOUS | 0.88474971 | NEUTRAL | 0.66884082 |
| E | 62 | A | NEUTRAL | 0.50824588 | NEUTRAL | 0.66884082 |
| E | 62 | K | DELETERIOUS | 0.73260309 | NEUTRAL | 0.66884082 |
| H | 67 | R | DELETERIOUS | 0.85822785 | DELETERIOUS | 0.59445019 |
| H | 67 | Q | DELETERIOUS | 0.67620995 | DELETERIOUS | 0.74491225 |
| N | 68 | D | DELETERIOUS | 0.67620995 | NEUTRAL | 0.66884082 |
| G | 70 | S | DELETERIOUS | 0.73260309 | DELETERIOUS | 0.74491225 |
| V | 71 | I | NEUTRAL | 0.7828765 | NEUTRAL | 0.66884082 |
| I | 76 | V | NEUTRAL | 0.68183996 | NEUTRAL | 0.66884082 |
| I | 76 | T | DELETERIOUS | 0.87523992 | DELETERIOUS | 0.74491225 |
| R | 82 | S | DELETERIOUS | 0.87523992 | DELETERIOUS | 0.74491225 |
| R | 82 | K | DELETERIOUS | 0.81731169 | DELETERIOUS | 0.74491225 |
| R | 82 | M | DELETERIOUS | 0.85822785 | DELETERIOUS | 0.74491225 |
| V | 83 | F | DELETERIOUS | 0.88474971 | DELETERIOUS | 0.74491225 |
| E | 84 | D | DELETERIOUS | 0.57795276 | NEUTRAL | 0.66884082 |
| E | 85 | G | DELETERIOUS | 0.87523992 | DELETERIOUS | 0.74491225 |
| F | 86 | L | DELETERIOUS | 0.85822785 | DELETERIOUS | 0.74491225 |
| L | 88 | F | DELETERIOUS | 0.67620995 | DELETERIOUS | 0.74491225 |
| M | 91 | I | DELETERIOUS | 0.85822785 | NEUTRAL | 0.66884082 |
| M | 91 | R | DELETERIOUS | 0.88474971 | DELETERIOUS | 0.74491225 |
| M | 91 | L | DELETERIOUS | 0.7733853 | NEUTRAL | 0.66884082 |
| M | 91 | T | DELETERIOUS | 0.87523992 | DELETERIOUS | 0.74491225 |
| W | 92 | G | DELETERIOUS | 0.87523992 | DELETERIOUS | 0.74491225 |
| W | 92 | R | DELETERIOUS | 0.87523992 | DELETERIOUS | 0.74491225 |
| K | 93 | E | DELETERIOUS | 0.73260309 | NEUTRAL | 0.66884082 |
| N | 96 | H | DELETERIOUS | 0.87523992 | DELETERIOUS | 0.74491225 |
| T | 98 | N | DELETERIOUS | 0.81731169 | DELETERIOUS | 0.74491225 |
| I | 99 | M | DELETERIOUS | 0.7733853 | DELETERIOUS | 0.74491225 |
| N | 101 | Y | DELETERIOUS | 0.88474971 | DELETERIOUS | 0.74491225 |
| T | 106 | M | DELETERIOUS | 0.73260309 | DELETERIOUS | 0.74491225 |
| V | 107 | I | NEUTRAL | 0.71871412 | NEUTRAL | 0.66884082 |
| F | 108 | V | DELETERIOUS | 0.81731169 | DELETERIOUS | 0.74491225 |
| R | 109 | K | DELETERIOUS | 0.85822785 | DELETERIOUS | 0.74491225 |
| I | 112 | V | NEUTRAL | 0.660879 | NEUTRAL | 0.66884082 |
| I | 113 | S | DELETERIOUS | 0.87523992 | DELETERIOUS | 0.74491225 |
| I | 113 | T | DELETERIOUS | 0.81731169 | DELETERIOUS | 0.74491225 |
| I | 117 | V | NEUTRAL | 0.83210379 | NEUTRAL | 0.66884082 |
| R | 119 | W | DELETERIOUS | 0.81731169 | DELETERIOUS | 0.74491225 |
| R | 119 | Q | DELETERIOUS | 0.87523992 | DELETERIOUS | 0.59445019 |
| S | 122 | N | DELETERIOUS | 0.57795276 | NEUTRAL | 0.66884082 |
| G | 123 | R | DELETERIOUS | 0.85822785 | NEUTRAL | 0.66884082 |
| V | 125 | L | DELETERIOUS | 0.58885542 | NEUTRAL | 0.66884082 |
| P | 127 | T | DELETERIOUS | 0.88474971 | DELETERIOUS | 0.74491225 |
| I | 130 | V | NEUTRAL | 0.71871412 | NEUTRAL | 0.66884082 |
| I | 130 | T | DELETERIOUS | 0.87523992 | DELETERIOUS | 0.59445019 |
| D | 137 | N | DELETERIOUS | 0.81731169 | DELETERIOUS | 0.74491225 |
| T | 142 | I | DELETERIOUS | 0.87523992 | NEUTRAL | 0.66884082 |
| P | 147 | S | DELETERIOUS | 0.67620995 | NEUTRAL | 0.66884082 |
| P | 149 | L | DELETERIOUS | 0.67620995 | DELETERIOUS | 0.59445019 |
| P | 149 | T | DELETERIOUS | 0.81731169 | NEUTRAL | 0.66884082 |
| G | 150 | R | DELETERIOUS | 0.7733853 | DELETERIOUS | 0.74491225 |
| V | 152 | G | DELETERIOUS | 0.81731169 | DELETERIOUS | 0.74491225 |
| E | 153 | Q | NEUTRAL | 0.68183996 | NEUTRAL | 0.66884082 |
| I | 154 | R | DELETERIOUS | 0.73260309 | DELETERIOUS | 0.74491225 |
| P | 158 | S | NEUTRAL | 0.55202703 | NEUTRAL | 0.66884082 |
| D | 160 | Y | DELETERIOUS | 0.87523992 | DELETERIOUS | 0.74491225 |
| G | 161 | R | DELETERIOUS | 0.7733853 | DELETERIOUS | 0.59445019 |
| T | 162 | S | NEUTRAL | 0.83210379 | NEUTRAL | 0.66884082 |
| T | 166 | I | NEUTRAL | 0.55202703 | NEUTRAL | 0.66884082 |
| E | 174 | D | NEUTRAL | 0.7828765 | NEUTRAL | 0.66884082 |
| G | 175 | D | DELETERIOUS | 0.87523992 | NEUTRAL | 0.66884082 |
| G | 177 | D | DELETERIOUS | 0.85822785 | DELETERIOUS | 0.59445019 |
| V | 178 | I | NEUTRAL | 0.71871412 | NEUTRAL | 0.66884082 |
| M | 180 | V | DELETERIOUS | 0.7733853 | NEUTRAL | 0.66884082 |
| M | 180 | L | NEUTRAL | 0.660879 | NEUTRAL | 0.66884082 |
| G | 181 | V | DELETERIOUS | 0.87523992 | DELETERIOUS | 0.74491225 |
| M | 182 | I | DELETERIOUS | 0.81731169 | DELETERIOUS | 0.59445019 |
| Y | 183 | H | DELETERIOUS | 0.7733853 | NEUTRAL | 0.66884082 |
| Y | 183 | C | DELETERIOUS | 0.88474971 | DELETERIOUS | 0.74491225 |
| I | 189 | V | NEUTRAL | 0.7828765 | NEUTRAL | 0.66884082 |
| E | 190 | K | DELETERIOUS | 0.7733853 | NEUTRAL | 0.66884082 |
| A | 193 | T | DELETERIOUS | 0.87523992 | DELETERIOUS | 0.74491225 |
| H | 194 | Y | DELETERIOUS | 0.73260309 | NEUTRAL | 0.66884082 |
| Q | 198 | R | DELETERIOUS | 0.67620995 | NEUTRAL | 0.66884082 |
| A | 200 | S | DELETERIOUS | 0.7733853 | NEUTRAL | 0.66884082 |
| L | 201 | V | DELETERIOUS | 0.7733853 | NEUTRAL | 0.66884082 |
| S | 202 | A | NEUTRAL | 0.7828765 | NEUTRAL | 0.66884082 |
| K | 203 | R | NEUTRAL | 0.71871412 | NEUTRAL | 0.66884082 |
| G | 204 | V | DELETERIOUS | 0.7733853 | NEUTRAL | 0.66884082 |
| L | 207 | W | DELETERIOUS | 0.7733853 | DELETERIOUS | 0.74491225 |
| Y | 208 | H | DELETERIOUS | 0.73260309 | DELETERIOUS | 0.74491225 |
| Y | 208 | C | DELETERIOUS | 0.81731169 | DELETERIOUS | 0.74491225 |
| T | 214 | S | DELETERIOUS | 0.87523992 | DELETERIOUS | 0.74491225 |
| K | 217 | R | DELETERIOUS | 0.67620995 | DELETERIOUS | 0.74491225 |
| Y | 219 | H | DELETERIOUS | 0.58885542 | DELETERIOUS | 0.59445019 |
| Y | 219 | C | DELETERIOUS | 0.81731169 | DELETERIOUS | 0.74491225 |
| D | 220 | G | DELETERIOUS | 0.81731169 | DELETERIOUS | 0.74491225 |
| R | 222 | H | DELETERIOUS | 0.73260309 | DELETERIOUS | 0.74491225 |
| R | 222 | C | DELETERIOUS | 0.81731169 | DELETERIOUS | 0.59445019 |
| E | 229 | V | DELETERIOUS | 0.85822785 | DELETERIOUS | 0.74491225 |
| Y | 231 | H | DELETERIOUS | 0.67620995 | DELETERIOUS | 0.59445019 |
| D | 232 | N | DELETERIOUS | 0.60798122 | NEUTRAL | 0.66884082 |
| K | 233 | M | NEUTRAL | 0.660879 | NEUTRAL | 0.66884082 |
| Y | 235 | C | DELETERIOUS | 0.85822785 | DELETERIOUS | 0.74491225 |
| K | 236 | Q | DELETERIOUS | 0.67620995 | NEUTRAL | 0.66884082 |
| Q | 238 | H | NEUTRAL | 0.50824588 | NEUTRAL | 0.66884082 |
| F | 239 | L | DELETERIOUS | 0.87523992 | DELETERIOUS | 0.59445019 |
| Q | 242 | H | NEUTRAL | 0.55202703 | NEUTRAL | 0.66884082 |
| K | 243 | E | DELETERIOUS | 0.81731169 | NEUTRAL | 0.66884082 |
| K | 243 | R | DELETERIOUS | 0.60798122 | DELETERIOUS | 0.59445019 |
| Y | 246 | H | DELETERIOUS | 0.87523992 | DELETERIOUS | 0.74491225 |
| D | 252 | N | DELETERIOUS | 0.85822785 | DELETERIOUS | 0.74491225 |
| A | 256 | V | DELETERIOUS | 0.87523992 | DELETERIOUS | 0.74491225 |
| A | 256 | S | DELETERIOUS | 0.87523992 | DELETERIOUS | 0.74491225 |
| K | 260 | N | DELETERIOUS | 0.85822785 | DELETERIOUS | 0.74491225 |
| G | 263 | E | DELETERIOUS | 0.88474971 | DELETERIOUS | 0.74491225 |
| G | 264 | A | DELETERIOUS | 0.87523992 | NEUTRAL | 0.66884082 |
| I | 266 | V | NEUTRAL | 0.89245476 | NEUTRAL | 0.66884082 |
| D | 273 | G | DELETERIOUS | 0.87523992 | DELETERIOUS | 0.59445019 |
| G | 274 | S | DELETERIOUS | 0.85822785 | DELETERIOUS | 0.74491225 |
| V | 276 | M | DELETERIOUS | 0.87523992 | DELETERIOUS | 0.74491225 |
| S | 278 | P | DELETERIOUS | 0.85822785 | DELETERIOUS | 0.74491225 |
| S | 278 | L | DELETERIOUS | 0.81731169 | DELETERIOUS | 0.59445019 |
| D | 279 | H | DELETERIOUS | 0.88474971 | DELETERIOUS | 0.74491225 |
| S | 280 | C | DELETERIOUS | 0.7733853 | NEUTRAL | 0.66884082 |
| S | 280 | F | DELETERIOUS | 0.60798122 | NEUTRAL | 0.66884082 |
| M | 291 | T | DELETERIOUS | 0.88474971 | DELETERIOUS | 0.74491225 |
| T | 292 | I | DELETERIOUS | 0.85822785 | DELETERIOUS | 0.74491225 |
| S | 293 | I | DELETERIOUS | 0.73260309 | DELETERIOUS | 0.74491225 |
| V | 294 | M | DELETERIOUS | 0.7733853 | DELETERIOUS | 0.59445019 |
| P | 298 | L | DELETERIOUS | 0.87523992 | DELETERIOUS | 0.74491225 |
| G | 300 | V | DELETERIOUS | 0.73260309 | DELETERIOUS | 0.74491225 |
| G | 300 | D | DELETERIOUS | 0.81731169 | DELETERIOUS | 0.59445019 |
| T | 302 | A | DELETERIOUS | 0.7733853 | NEUTRAL | 0.66884082 |
| E | 304 | G | DELETERIOUS | 0.85822785 | DELETERIOUS | 0.59445019 |
| A | 305 | T | DELETERIOUS | 0.81731169 | NEUTRAL | 0.66884082 |
| E | 306 | A | DELETERIOUS | 0.85822785 | DELETERIOUS | 0.74491225 |
| H | 309 | R | DELETERIOUS | 0.87523992 | DELETERIOUS | 0.59445019 |
| H | 309 | Q | DELETERIOUS | 0.87523992 | DELETERIOUS | 0.74491225 |
| G | 310 | R | DELETERIOUS | 0.85822785 | DELETERIOUS | 0.74491225 |
| R | 314 | H | DELETERIOUS | 0.85822785 | DELETERIOUS | 0.74491225 |
| R | 314 | C | DELETERIOUS | 0.85822785 | DELETERIOUS | 0.74491225 |
| H | 315 | D | DELETERIOUS | 0.88474971 | DELETERIOUS | 0.74491225 |
| R | 317 | H | DELETERIOUS | 0.87523992 | DELETERIOUS | 0.59445019 |
| R | 317 | C | DELETERIOUS | 0.87523992 | DELETERIOUS | 0.74491225 |
| R | 317 | L | DELETERIOUS | 0.88474971 | DELETERIOUS | 0.74491225 |
| M | 318 | T | DELETERIOUS | 0.58885542 | NEUTRAL | 0.66884082 |
| K | 321 | E | DELETERIOUS | 0.7733853 | DELETERIOUS | 0.74491225 |
| T | 325 | M | DELETERIOUS | 0.87523992 | DELETERIOUS | 0.74491225 |
| S | 326 | P | DELETERIOUS | 0.85822785 | DELETERIOUS | 0.74491225 |
| T | 327 | I | DELETERIOUS | 0.88474971 | DELETERIOUS | 0.74491225 |
| N | 328 | S | DELETERIOUS | 0.88474971 | DELETERIOUS | 0.74491225 |
| N | 328 | K | DELETERIOUS | 0.88474971 | DELETERIOUS | 0.74491225 |
| I | 330 | T | DELETERIOUS | 0.87523992 | DELETERIOUS | 0.74491225 |
| I | 333 | S | DELETERIOUS | 0.88474971 | DELETERIOUS | 0.74491225 |
| A | 335 | S | DELETERIOUS | 0.88474971 | NEUTRAL | 0.66884082 |
| R | 338 | S | DELETERIOUS | 0.88474971 | NEUTRAL | 0.66884082 |
| G | 339 | R | DELETERIOUS | 0.87523992 | DELETERIOUS | 0.74491225 |
| A | 341 | V | DELETERIOUS | 0.58885542 | NEUTRAL | 0.66884082 |
| A | 341 | P | DELETERIOUS | 0.87523992 | DELETERIOUS | 0.74491225 |
| A | 341 | D | DELETERIOUS | 0.87523992 | NEUTRAL | 0.66884082 |
| L | 346 | P | DELETERIOUS | 0.87523992 | DELETERIOUS | 0.74491225 |
| E | 351 | G | DELETERIOUS | 0.58885542 | NEUTRAL | 0.66884082 |
| E | 351 | K | NEUTRAL | 0.50824588 | NEUTRAL | 0.66884082 |
| A | 353 | G | NEUTRAL | 0.68183996 | NEUTRAL | 0.66884082 |
| A | 353 | D | DELETERIOUS | 0.58885542 | NEUTRAL | 0.66884082 |
| F | 354 | V | NEUTRAL | 0.7828765 | NEUTRAL | 0.66884082 |
| F | 354 | L | NEUTRAL | 0.7828765 | NEUTRAL | 0.66884082 |
| A | 356 | V | DELETERIOUS | 0.81731169 | NEUTRAL | 0.66884082 |
| S | 363 | A | NEUTRAL | 0.58230958 | NEUTRAL | 0.66884082 |
| I | 364 | V | NEUTRAL | 0.83210379 | NEUTRAL | 0.66884082 |
| I | 364 | L | DELETERIOUS | 0.7733853 | NEUTRAL | 0.66884082 |
| E | 365 | V | DELETERIOUS | 0.67620995 | DELETERIOUS | 0.59445019 |
| I | 367 | V | NEUTRAL | 0.89245476 | NEUTRAL | 0.66884082 |
| I | 367 | T | DELETERIOUS | 0.58885542 | DELETERIOUS | 0.59445019 |
| A | 369 | V | DELETERIOUS | 0.57795276 | NEUTRAL | 0.66884082 |
| G | 370 | V | DELETERIOUS | 0.87523992 | DELETERIOUS | 0.74491225 |
| M | 372 | T | DELETERIOUS | 0.87523992 | DELETERIOUS | 0.74491225 |
| T | 373 | I | DELETERIOUS | 0.85822785 | DELETERIOUS | 0.74491225 |
| T | 373 | N | DELETERIOUS | 0.81731169 | DELETERIOUS | 0.74491225 |
| K | 374 | E | DELETERIOUS | 0.85822785 | DELETERIOUS | 0.59445019 |
| A | 378 | V | NEUTRAL | 0.55202703 | NEUTRAL | 0.66884082 |
| A | 378 | G | NEUTRAL | 0.58230958 | NEUTRAL | 0.66884082 |
| L | 383 | F | NEUTRAL | 0.55202703 | NEUTRAL | 0.66884082 |
| P | 384 | L | NEUTRAL | 0.50824588 | NEUTRAL | 0.66884082 |
| N | 385 | S | DELETERIOUS | 0.60798122 | NEUTRAL | 0.66884082 |
| N | 385 | D | NEUTRAL | 0.68183996 | NEUTRAL | 0.66884082 |
| R | 388 | H | DELETERIOUS | 0.85822785 | DELETERIOUS | 0.59445019 |
| R | 388 | C | DELETERIOUS | 0.88474971 | DELETERIOUS | 0.74491225 |
| M | 398 | I | NEUTRAL | 0.68183996 | NEUTRAL | 0.66884082 |
| D | 399 | H | DELETERIOUS | 0.7733853 | DELETERIOUS | 0.59445019 |
| D | 399 | G | DELETERIOUS | 0.7733853 | NEUTRAL | 0.66884082 |
| L | 401 | P | DELETERIOUS | 0.81731169 | DELETERIOUS | 0.74491225 |
| G | 402 | E | NEUTRAL | 0.660879 | NEUTRAL | 0.66884082 |
| L | 405 | V | DELETERIOUS | 0.67620995 | NEUTRAL | 0.66884082 |
| K | 408 | Q | NEUTRAL | 0.83210379 | NEUTRAL | 0.66884082 |

**Table 3. Analysis of nSNPS using different servers.**

| **Wild residue** | **Position** | **Target residue** | **PolyPhen-2 prediction** | **PolyPhen-2 expected accuracy** | **SIFT prediction** | **SIFT expected accuracy** |
| --- | --- | --- | --- | --- | --- | --- |
| S | 2 | P | NEUTRAL | 0.74074074 | DELETERIOUS | 0.42969871 |
| K | 4 | Q | DELETERIOUS | 0.43123393 | NEUTRAL | 0.76067616 |
| S | 6 | G | NEUTRAL | 0.74974975 | NEUTRAL | 0.76067616 |
| G | 8 | S | DELETERIOUS | 0.39845758 | DELETERIOUS | 0.52778524 |
| E | 12 | Q | DELETERIOUS | 0.63431877 | DELETERIOUS | 0.79280784 |
| Q | 14 | R | NEUTRAL | 0.69369369 | DELETERIOUS | 0.52778524 |
| G | 15 | E | DELETERIOUS | 0.67524116 | DELETERIOUS | 0.79280784 |
| D | 16 | H | DELETERIOUS | 0.81142888 | DELETERIOUS | 0.79280784 |
| T | 19 | A | NEUTRAL | 0.60960961 | DELETERIOUS | 0.45949821 |
| R | 20 | Q | DELETERIOUS | 0.60089974 | DELETERIOUS | 0.45232975 |
| R | 20 | L | DELETERIOUS | 0.67524116 | DELETERIOUS | 0.79280784 |
| I | 22 | V | NEUTRAL | 0.7027027 | DELETERIOUS | 0.79280784 |
| W | 23 | C | DELETERIOUS | 0.60089974 | DELETERIOUS | 0.79280784 |
| K | 27 | Q | NEUTRAL | 0.60960961 | DELETERIOUS | 0.79280784 |
| L | 30 | V | NEUTRAL | 0.60960961 | DELETERIOUS | 0.79280784 |
| L | 30 | I | DELETERIOUS | 0.45308483 | DELETERIOUS | 0.52778524 |
| I | 31 | V | NEUTRAL | 0.74074074 | DELETERIOUS | 0.79280784 |
| F | 32 | V | NEUTRAL | 0.74974975 | DELETERIOUS | 0.45949821 |
| P | 33 | S | DELETERIOUS | 0.47429306 | DELETERIOUS | 0.52778524 |
| Y | 34 | C | DELETERIOUS | 0.67524116 | DELETERIOUS | 0.79280784 |
| V | 35 | A | DELETERIOUS | 0.39845758 | DELETERIOUS | 0.79280784 |
| V | 35 | M | DELETERIOUS | 0.45308483 | DELETERIOUS | 0.52778524 |
| D | 38 | N | NEUTRAL | 0.78928929 | NEUTRAL | 0.67141585 |
| S | 41 | G | NEUTRAL | 0.67667668 | DELETERIOUS | 0.79280784 |
| Y | 42 | C | DELETERIOUS | 0.81142888 | DELETERIOUS | 0.79280784 |
| D | 43 | A | DELETERIOUS | 0.67524116 | DELETERIOUS | 0.79280784 |
| R | 49 | H | DELETERIOUS | 0.43123393 | DELETERIOUS | 0.52778524 |
| R | 49 | C | DELETERIOUS | 0.47429306 | DELETERIOUS | 0.45232975 |
| R | 49 | P | DELETERIOUS | 0.39845758 | DELETERIOUS | 0.79280784 |
| A | 51 | G | NEUTRAL | 0.72472472 | DELETERIOUS | 0.52778524 |
| D | 54 | N | DELETERIOUS | 0.81142888 | DELETERIOUS | 0.79280784 |
| Q | 55 | P | DELETERIOUS | 0.39845758 | DELETERIOUS | 0.79280784 |
| A | 61 | T | NEUTRAL | 0.7027027 | DELETERIOUS | 0.79280784 |
| E | 62 | A | NEUTRAL | 0.78928929 | NEUTRAL | 0.64501779 |
| E | 62 | K | NEUTRAL | 0.78928929 | NEUTRAL | 0.7366548 |
| H | 67 | R | NEUTRAL | 0.69369369 | DELETERIOUS | 0.79280784 |
| H | 67 | Q | NEUTRAL | 0.69369369 | DELETERIOUS | 0.79280784 |
| N | 68 | D | NEUTRAL | 0.78928929 | DELETERIOUS | 0.45949821 |
| G | 70 | S | DELETERIOUS | 0.60089974 | DELETERIOUS | 0.79280784 |
| V | 71 | I | NEUTRAL | 0.74074074 | NEUTRAL | 0.89670526 |
| I | 76 | V | DELETERIOUS | 0.50257069 | DELETERIOUS | 0.79280784 |
| I | 76 | T | DELETERIOUS | 0.81142888 | DELETERIOUS | 0.79280784 |
| R | 82 | S | DELETERIOUS | 0.64717224 | DELETERIOUS | 0.52778524 |
| R | 82 | K | DELETERIOUS | 0.67524116 | DELETERIOUS | 0.79280784 |
| R | 82 | M | DELETERIOUS | 0.81142888 | DELETERIOUS | 0.79280784 |
| V | 83 | F | DELETERIOUS | 0.64974293 | DELETERIOUS | 0.79280784 |
| E | 84 | D | NEUTRAL | 0.8730899 | DELETERIOUS | 0.52778524 |
| E | 85 | G | DELETERIOUS | 0.64974293 | DELETERIOUS | 0.79280784 |
| F | 86 | L | DELETERIOUS | 0.59318766 | DELETERIOUS | 0.79280784 |
| L | 88 | F | DELETERIOUS | 0.81142888 | DELETERIOUS | 0.79280784 |
| M | 91 | I | DELETERIOUS | 0.47429306 | DELETERIOUS | 0.79280784 |
| M | 91 | R | DELETERIOUS | 0.81142888 | DELETERIOUS | 0.79280784 |
| M | 91 | L | DELETERIOUS | 0.39845758 | DELETERIOUS | 0.79280784 |
| M | 91 | T | DELETERIOUS | 0.67524116 | DELETERIOUS | 0.79280784 |
| W | 92 | G | DELETERIOUS | 0.81142888 | DELETERIOUS | 0.79280784 |
| W | 92 | R | DELETERIOUS | 0.81142888 | DELETERIOUS | 0.79280784 |
| K | 93 | E | NEUTRAL | 0.71171171 | DELETERIOUS | 0.79280784 |
| N | 96 | H | DELETERIOUS | 0.81142888 | DELETERIOUS | 0.79280784 |
| T | 98 | N | DELETERIOUS | 0.67524116 | DELETERIOUS | 0.79280784 |
| I | 99 | M | DELETERIOUS | 0.64974293 | DELETERIOUS | 0.79280784 |
| N | 101 | Y | DELETERIOUS | 0.81142888 | DELETERIOUS | 0.79280784 |
| T | 106 | M | DELETERIOUS | 0.81142888 | DELETERIOUS | 0.79280784 |
| V | 107 | I | NEUTRAL | 0.63363363 | NEUTRAL | 0.67141585 |
| F | 108 | V | DELETERIOUS | 0.81142888 | DELETERIOUS | 0.79280784 |
| R | 109 | K | DELETERIOUS | 0.81142888 | DELETERIOUS | 0.79280784 |
| I | 112 | V | DELETERIOUS | 0.47429306 | DELETERIOUS | 0.52778524 |
| I | 113 | S | DELETERIOUS | 0.56233933 | DELETERIOUS | 0.79280784 |
| I | 113 | T | DELETERIOUS | 0.47429306 | DELETERIOUS | 0.79280784 |
| I | 117 | V | NEUTRAL | 0.78928929 | NEUTRAL | 0.82295374 |
| R | 119 | W | DELETERIOUS | 0.55077121 | DELETERIOUS | 0.45232975 |
| R | 119 | Q | DELETERIOUS | 0.50257069 | DELETERIOUS | 0.79280784 |
| S | 122 | N | NEUTRAL | 0.8730899 | NEUTRAL | 0.52894034 |
| G | 123 | R | NEUTRAL | 0.60960961 | DELETERIOUS | 0.79280784 |
| V | 125 | L | NEUTRAL | 0.8730899 | DELETERIOUS | 0.52778524 |
| P | 127 | T | DELETERIOUS | 0.55077121 | DELETERIOUS | 0.79280784 |
| I | 130 | V | NEUTRAL | 0.8730899 | NEUTRAL | 0.67141585 |
| I | 130 | T | NEUTRAL | 0.70240481 | DELETERIOUS | 0.79280784 |
| D | 137 | N | DELETERIOUS | 0.81142888 | DELETERIOUS | 0.79280784 |
| T | 142 | I | NEUTRAL | 0.67635271 | DELETERIOUS | 0.52778524 |
| P | 147 | S | NEUTRAL | 0.72472472 | NEUTRAL | 0.60819234 |
| P | 149 | L | DELETERIOUS | 0.39845758 | DELETERIOUS | 0.79280784 |
| P | 149 | T | NEUTRAL | 0.72272272 | DELETERIOUS | 0.79280784 |
| G | 150 | R | DELETERIOUS | 0.81142888 | DELETERIOUS | 0.79280784 |
| V | 152 | G | DELETERIOUS | 0.59318766 | DELETERIOUS | 0.79280784 |
| E | 153 | Q | NEUTRAL | 0.74074074 | NEUTRAL | 0.64501779 |
| I | 154 | R | DELETERIOUS | 0.40745501 | DELETERIOUS | 0.79280784 |
| P | 158 | S | NEUTRAL | 0.63363363 | NEUTRAL | 0.64501779 |
| D | 160 | Y | DELETERIOUS | 0.54177378 | DELETERIOUS | 0.79280784 |
| G | 161 | R | NEUTRAL | 0.62762763 | DELETERIOUS | 0.79280784 |
| T | 162 | S | NEUTRAL | 0.8730899 | NEUTRAL | 0.89670526 |
| T | 166 | I | NEUTRAL | 0.78928929 | NEUTRAL | 0.60819234 |
| E | 174 | D | NEUTRAL | 0.8730899 | NEUTRAL | 0.76135352 |
| G | 175 | D | NEUTRAL | 0.8730899 | NEUTRAL | 0.64501779 |
| G | 177 | D | DELETERIOUS | 0.81142888 | DELETERIOUS | 0.79280784 |
| V | 178 | I | NEUTRAL | 0.72745491 | NEUTRAL | 0.6841637 |
| M | 180 | V | NEUTRAL | 0.67667668 | DELETERIOUS | 0.52778524 |
| M | 180 | L | NEUTRAL | 0.8730899 | NEUTRAL | 0.79073909 |
| G | 181 | V | DELETERIOUS | 0.39845758 | DELETERIOUS | 0.79280784 |
| M | 182 | I | DELETERIOUS | 0.45308483 | DELETERIOUS | 0.79280784 |
| Y | 183 | H | NEUTRAL | 0.60960961 | DELETERIOUS | 0.79280784 |
| Y | 183 | C | DELETERIOUS | 0.81142888 | DELETERIOUS | 0.79280784 |
| I | 189 | V | NEUTRAL | 0.69369369 | DELETERIOUS | 0.45949821 |
| E | 190 | K | NEUTRAL | 0.8730899 | NEUTRAL | 0.76224399 |
| A | 193 | T | DELETERIOUS | 0.67524116 | DELETERIOUS | 0.79280784 |
| H | 194 | Y | NEUTRAL | 0.74074074 | NEUTRAL | 0.67141585 |
| Q | 198 | R | NEUTRAL | 0.8730899 | NEUTRAL | 0.6841637 |
| A | 200 | S | NEUTRAL | 0.68268268 | NEUTRAL | 0.60819234 |
| L | 201 | V | NEUTRAL | 0.72745491 | DELETERIOUS | 0.79280784 |
| S | 202 | A | NEUTRAL | 0.8730899 | NEUTRAL | 0.75800712 |
| K | 203 | R | NEUTRAL | 0.78928929 | NEUTRAL | 0.75800712 |
| G | 204 | V | NEUTRAL | 0.74974975 | DELETERIOUS | 0.52778524 |
| L | 207 | W | DELETERIOUS | 0.81142888 | DELETERIOUS | 0.79280784 |
| Y | 208 | H | DELETERIOUS | 0.81142888 | DELETERIOUS | 0.79280784 |
| Y | 208 | C | DELETERIOUS | 0.81142888 | DELETERIOUS | 0.79280784 |
| T | 214 | S | DELETERIOUS | 0.63431877 | DELETERIOUS | 0.79280784 |
| K | 217 | R | DELETERIOUS | 0.81142888 | DELETERIOUS | 0.79280784 |
| Y | 219 | H | DELETERIOUS | 0.54177378 | DELETERIOUS | 0.45949821 |
| Y | 219 | C | DELETERIOUS | 0.81142888 | DELETERIOUS | 0.79280784 |
| D | 220 | G | DELETERIOUS | 0.67524116 | DELETERIOUS | 0.79280784 |
| R | 222 | H | DELETERIOUS | 0.63431877 | DELETERIOUS | 0.79280784 |
| R | 222 | C | DELETERIOUS | 0.45308483 | DELETERIOUS | 0.45232975 |
| E | 229 | V | NEUTRAL | 0.63363363 | DELETERIOUS | 0.79280784 |
| Y | 231 | H | DELETERIOUS | 0.39845758 | DELETERIOUS | 0.45949821 |
| D | 232 | N | NEUTRAL | 0.8730899 | NEUTRAL | 0.67141585 |
| K | 233 | M | NEUTRAL | 0.76252505 | DELETERIOUS | 0.52778524 |
| Y | 235 | C | DELETERIOUS | 0.81142888 | DELETERIOUS | 0.79280784 |
| K | 236 | Q | NEUTRAL | 0.76252505 | DELETERIOUS | 0.45949821 |
| Q | 238 | H | NEUTRAL | 0.8730899 | DELETERIOUS | 0.52778524 |
| F | 239 | L | DELETERIOUS | 0.47429306 | DELETERIOUS | 0.79280784 |
| Q | 242 | H | NEUTRAL | 0.8730899 | NEUTRAL | 0.75800712 |
| K | 243 | E | NEUTRAL | 0.76252505 | DELETERIOUS | 0.52778524 |
| K | 243 | R | NEUTRAL | 0.67635271 | DELETERIOUS | 0.79280784 |
| Y | 246 | H | DELETERIOUS | 0.81142888 | DELETERIOUS | 0.79280784 |
| D | 252 | N | NEUTRAL | 0.60960961 | DELETERIOUS | 0.79280784 |
| A | 256 | V | DELETERIOUS | 0.81142888 | DELETERIOUS | 0.79280784 |
| A | 256 | S | DELETERIOUS | 0.81142888 | DELETERIOUS | 0.79280784 |
| K | 260 | N | DELETERIOUS | 0.81142888 | DELETERIOUS | 0.79280784 |
| G | 263 | E | DELETERIOUS | 0.81142888 | DELETERIOUS | 0.79280784 |
| G | 264 | A | NEUTRAL | 0.60960961 | DELETERIOUS | 0.45232975 |
| I | 266 | V | NEUTRAL | 0.8730899 | NEUTRAL | 0.89670526 |
| D | 273 | G | DELETERIOUS | 0.55077121 | DELETERIOUS | 0.79280784 |
| G | 274 | S | DELETERIOUS | 0.81142888 | DELETERIOUS | 0.79280784 |
| V | 276 | M | DELETERIOUS | 0.81142888 | DELETERIOUS | 0.79280784 |
| S | 278 | P | DELETERIOUS | 0.81142888 | DELETERIOUS | 0.79280784 |
| S | 278 | L | DELETERIOUS | 0.63431877 | DELETERIOUS | 0.52778524 |
| D | 279 | H | DELETERIOUS | 0.81142888 | DELETERIOUS | 0.79280784 |
| S | 280 | C | NEUTRAL | 0.70770771 | NEUTRAL | 0.66429207 |
| S | 280 | F | NEUTRAL | 0.72272272 | NEUTRAL | 0.64501779 |
| M | 291 | T | DELETERIOUS | 0.81142888 | DELETERIOUS | 0.79280784 |
| T | 292 | I | DELETERIOUS | 0.81142888 | DELETERIOUS | 0.79280784 |
| S | 293 | I | DELETERIOUS | 0.81142888 | DELETERIOUS | 0.79280784 |
| V | 294 | M | DELETERIOUS | 0.60089974 | DELETERIOUS | 0.79280784 |
| P | 298 | L | DELETERIOUS | 0.40745501 | DELETERIOUS | 0.79280784 |
| G | 300 | V | DELETERIOUS | 0.81142888 | DELETERIOUS | 0.79280784 |
| G | 300 | D | DELETERIOUS | 0.43123393 | DELETERIOUS | 0.79280784 |
| T | 302 | A | NEUTRAL | 0.74974975 | DELETERIOUS | 0.79280784 |
| E | 304 | G | NEUTRAL | 0.7027027 | DELETERIOUS | 0.79280784 |
| A | 305 | T | NEUTRAL | 0.70770771 | DELETERIOUS | 0.52778524 |
| E | 306 | A | DELETERIOUS | 0.67524116 | DELETERIOUS | 0.79280784 |
| H | 309 | R | DELETERIOUS | 0.43123393 | DELETERIOUS | 0.79280784 |
| H | 309 | Q | DELETERIOUS | 0.67524116 | DELETERIOUS | 0.52778524 |
| G | 310 | R | DELETERIOUS | 0.54177378 | DELETERIOUS | 0.45232975 |
| R | 314 | H | DELETERIOUS | 0.81142888 | DELETERIOUS | 0.79280784 |
| R | 314 | C | DELETERIOUS | 0.81142888 | DELETERIOUS | 0.52778524 |
| H | 315 | D | DELETERIOUS | 0.81142888 | DELETERIOUS | 0.79280784 |
| R | 317 | H | DELETERIOUS | 0.60089974 | DELETERIOUS | 0.42969871 |
| R | 317 | C | DELETERIOUS | 0.81142888 | DELETERIOUS | 0.79280784 |
| R | 317 | L | DELETERIOUS | 0.64974293 | DELETERIOUS | 0.79280784 |
| M | 318 | T | NEUTRAL | 0.70770771 | NEUTRAL | 0.67141585 |
| K | 321 | E | NEUTRAL | 0.78928929 | DELETERIOUS | 0.52778524 |
| T | 325 | M | DELETERIOUS | 0.81142888 | DELETERIOUS | 0.79280784 |
| S | 326 | P | DELETERIOUS | 0.81142888 | DELETERIOUS | 0.79280784 |
| T | 327 | I | DELETERIOUS | 0.81142888 | DELETERIOUS | 0.79280784 |
| N | 328 | S | DELETERIOUS | 0.67524116 | DELETERIOUS | 0.79280784 |
| N | 328 | K | DELETERIOUS | 0.81142888 | DELETERIOUS | 0.52778524 |
| I | 330 | T | DELETERIOUS | 0.47429306 | DELETERIOUS | 0.79280784 |
| I | 333 | S | DELETERIOUS | 0.81142888 | DELETERIOUS | 0.79280784 |
| A | 335 | S | DELETERIOUS | 0.45308483 | DELETERIOUS | 0.79280784 |
| R | 338 | S | DELETERIOUS | 0.39845758 | DELETERIOUS | 0.52778524 |
| G | 339 | R | DELETERIOUS | 0.81142888 | DELETERIOUS | 0.79280784 |
| A | 341 | V | NEUTRAL | 0.72745491 | NEUTRAL | 0.66429207 |
| A | 341 | P | DELETERIOUS | 0.40745501 | NEUTRAL | 0.52894034 |
| A | 341 | D | NEUTRAL | 0.74074074 | NEUTRAL | 0.71326803 |
| L | 346 | P | DELETERIOUS | 0.67524116 | DELETERIOUS | 0.79280784 |
| E | 351 | G | NEUTRAL | 0.8730899 | DELETERIOUS | 0.45949821 |
| E | 351 | K | NEUTRAL | 0.8730899 | NEUTRAL | 0.75690116 |
| A | 353 | G | NEUTRAL | 0.8730899 | NEUTRAL | 0.76936776 |
| A | 353 | D | NEUTRAL | 0.8730899 | NEUTRAL | 0.79073909 |
| F | 354 | V | NEUTRAL | 0.8730899 | NEUTRAL | 0.75800712 |
| F | 354 | L | NEUTRAL | 0.8730899 | NEUTRAL | 0.74888691 |
| A | 356 | V | NEUTRAL | 0.60960961 | NEUTRAL | 0.60819234 |
| S | 363 | A | NEUTRAL | 0.8730899 | DELETERIOUS | 0.79280784 |
| I | 364 | V | NEUTRAL | 0.8730899 | NEUTRAL | 0.83526269 |
| I | 364 | L | NEUTRAL | 0.8730899 | DELETERIOUS | 0.52778524 |
| E | 365 | V | NEUTRAL | 0.64364364 | DELETERIOUS | 0.52778524 |
| I | 367 | V | NEUTRAL | 0.8730899 | NEUTRAL | 0.89670526 |
| I | 367 | T | DELETERIOUS | 0.55077121 | DELETERIOUS | 0.79280784 |
| A | 369 | V | NEUTRAL | 0.8730899 | DELETERIOUS | 0.45949821 |
| G | 370 | V | DELETERIOUS | 0.63431877 | DELETERIOUS | 0.79280784 |
| M | 372 | T | DELETERIOUS | 0.60089974 | DELETERIOUS | 0.79280784 |
| T | 373 | I | DELETERIOUS | 0.81142888 | DELETERIOUS | 0.79280784 |
| T | 373 | N | DELETERIOUS | 0.63431877 | DELETERIOUS | 0.79280784 |
| K | 374 | E | DELETERIOUS | 0.43123393 | DELETERIOUS | 0.45949821 |
| A | 378 | V | NEUTRAL | 0.74974975 | NEUTRAL | 0.76936776 |
| A | 378 | G | NEUTRAL | 0.8730899 | NEUTRAL | 0.75690116 |
| L | 383 | F | NEUTRAL | 0.76252505 | NEUTRAL | 0.67141585 |
| P | 384 | L | NEUTRAL | 0.71171171 | DELETERIOUS | 0.45949821 |
| N | 385 | S | NEUTRAL | 0.74974975 | NEUTRAL | 0.64501779 |
| N | 385 | D | NEUTRAL | 0.8730899 | NEUTRAL | 0.72662511 |
| R | 388 | H | NEUTRAL | 0.70240481 | DELETERIOUS | 0.52778524 |
| R | 388 | C | DELETERIOUS | 0.64717224 | DELETERIOUS | 0.52778524 |
| M | 398 | I | NEUTRAL | 0.8730899 | NEUTRAL | 0.79073909 |
| D | 399 | H | NEUTRAL | 0.67667668 | DELETERIOUS | 0.52778524 |
| D | 399 | G | NEUTRAL | 0.74074074 | DELETERIOUS | 0.45232975 |
| L | 401 | P | DELETERIOUS | 0.64717224 | DELETERIOUS | 0.79280784 |
| G | 402 | E | NEUTRAL | 0.8730899 | NEUTRAL | 0.74888691 |
| L | 405 | V | NEUTRAL | 0.72272272 | DELETERIOUS | 0.52778524 |
| K | 408 | Q | NEUTRAL | 0.72272272 | NEUTRAL | 0.70614426 |

**Table 4. Analysis of nSNPS using different servers.**

| **Wild residue** | **Position** | **Target residue** | **SNAP prediction** | **SNAP expected accuracy** | **PANTHER prediction** | **PANTHER expected accuracy** |
| --- | --- | --- | --- | --- | --- | --- |
| S | 2 | P | NEUTRAL | 0.83058824 | NEUTRAL | 0.48435689 |
| K | 4 | Q | DELETERIOUS | 0.62208185 | DELETERIOUS | 0.568 |
| S | 6 | G | NEUTRAL | 0.66524217 | NEUTRAL | 0.6400463 |
| G | 8 | S | NEUTRAL | 0.83058824 | NEUTRAL | 0.66550926 |
| E | 12 | Q | DELETERIOUS | 0.72038776 | DELETERIOUS | 0.68662675 |
| Q | 14 | R | NEUTRAL | 0.58359402 | NEUTRAL | 0.56199305 |
| G | 15 | E | DELETERIOUS | 0.88519637 | DELETERIOUS | 0.78 |
| D | 16 | H | DELETERIOUS | 0.88519637 | DELETERIOUS | 0.78 |
| T | 19 | A | NEUTRAL | 0.58359402 | NEUTRAL | 0.48319815 |
| R | 20 | Q | NEUTRAL | 0.58359402 | DELETERIOUS | 0.654 |
| R | 20 | L | DELETERIOUS | 0.62208185 | DELETERIOUS | 0.71856287 |
| I | 22 | V | NEUTRAL | 0.66524217 | NEUTRAL | 0.48032407 |
| W | 23 | C | DELETERIOUS | 0.62208185 | DELETERIOUS | 0.8742515 |
| K | 27 | Q | NEUTRAL | 0.50014676 | DELETERIOUS | 0.568 |
| L | 30 | V | NEUTRAL | 0.61167883 | DELETERIOUS | 0.66067864 |
| L | 30 | I | NEUTRAL | 0.55439739 | DELETERIOUS | 0.60678643 |
| I | 31 | V | NEUTRAL | 0.70878378 | NEUTRAL | 0.55671296 |
| F | 32 | V | NEUTRAL | 0.58359402 | NEUTRAL | 0.48611111 |
| P | 33 | S | NEUTRAL | 0.50014676 | DELETERIOUS | 0.74451098 |
| Y | 34 | C | DELETERIOUS | 0.55551884 | DELETERIOUS | 0.66267465 |
| V | 35 | A | DELETERIOUS | 0.55551884 | NEUTRAL | 0.48611111 |
| V | 35 | M | NEUTRAL | 0.61167883 | NEUTRAL | 0.63310185 |
| D | 38 | N | NEUTRAL | 0.66524217 | NEUTRAL | 0.54808806 |
| S | 41 | G | NEUTRAL | 0.50014676 | DELETERIOUS | 0.78 |
| Y | 42 | C | DELETERIOUS | 0.84845361 | DELETERIOUS | 0.74451098 |
| D | 43 | A | DELETERIOUS | 0.80510276 | DELETERIOUS | 0.74451098 |
| R | 49 | H | DELETERIOUS | 0.72038776 | DELETERIOUS | 0.84231537 |
| R | 49 | C | DELETERIOUS | 0.80510276 | DELETERIOUS | 0.74451098 |
| R | 49 | P | DELETERIOUS | 0.88519637 | NEUTRAL | 0.48435689 |
| A | 51 | G | NEUTRAL | 0.66524217 | DELETERIOUS | 0.744 |
| D | 54 | N | NEUTRAL | 0.58359402 | DELETERIOUS | 0.71457086 |
| Q | 55 | P | NEUTRAL | 0.50014676 | DELETERIOUS | 0.69860279 |
| A | 61 | T | NEUTRAL | 0.58359402 | NEUTRAL | 0.55671296 |
| E | 62 | A | NEUTRAL | 0.61167883 | NEUTRAL | 0.63541667 |
| E | 62 | K | NEUTRAL | 0.70878378 | NEUTRAL | 0.6400463 |
| H | 67 | R | DELETERIOUS | 0.62208185 | NEUTRAL | 0.6400463 |
| H | 67 | Q | DELETERIOUS | 0.62208185 | NEUTRAL | 0.55671296 |
| N | 68 | D | NEUTRAL | 0.55439739 | DELETERIOUS | 0.568 |
| G | 70 | S | NEUTRAL | 0.50014676 | NEUTRAL | 0.67091541 |
| V | 71 | I | NEUTRAL | 0.76823399 | NEUTRAL | 0.47222222 |
| I | 76 | V | NEUTRAL | 0.50014676 | DELETERIOUS | 0.744 |
| I | 76 | T | DELETERIOUS | 0.62208185 | DELETERIOUS | 0.76 |
| R | 82 | S | DELETERIOUS | 0.72038776 | DELETERIOUS | 0.69 |
| R | 82 | K | DELETERIOUS | 0.62208185 | DELETERIOUS | 0.84231537 |
| R | 82 | M | DELETERIOUS | 0.72038776 | DELETERIOUS | 0.71856287 |
| V | 83 | F | DELETERIOUS | 0.72038776 | NEUTRAL | 0.48032407 |
| E | 84 | D | NEUTRAL | 0.66524217 | DELETERIOUS | 0.74451098 |
| E | 85 | G | DELETERIOUS | 0.72038776 | DELETERIOUS | 0.678 |
| F | 86 | L | NEUTRAL | 0.58359402 | DELETERIOUS | 0.71457086 |
| L | 88 | F | DELETERIOUS | 0.55551884 | DELETERIOUS | 0.61 |
| M | 91 | I | DELETERIOUS | 0.55551884 | DELETERIOUS | 0.69860279 |
| M | 91 | R | DELETERIOUS | 0.72038776 | DELETERIOUS | 0.56686627 |
| M | 91 | L | NEUTRAL | 0.55439739 | DELETERIOUS | 0.66067864 |
| M | 91 | T | DELETERIOUS | 0.72038776 | DELETERIOUS | 0.74451098 |
| W | 92 | G | DELETERIOUS | 0.84845361 | DELETERIOUS | 0.78 |
| W | 92 | R | DELETERIOUS | 0.84845361 | DELETERIOUS | 0.61 |
| K | 93 | E | DELETERIOUS | 0.55551884 | DELETERIOUS | 0.78 |
| N | 96 | H | DELETERIOUS | 0.80510276 | DELETERIOUS | 0.76 |
| T | 98 | N | DELETERIOUS | 0.62208185 | DELETERIOUS | 0.71856287 |
| I | 99 | M | NEUTRAL | 0.66524217 | DELETERIOUS | 0.76646707 |
| N | 101 | Y | DELETERIOUS | 0.62208185 | DELETERIOUS | 0.76 |
| T | 106 | M | DELETERIOUS | 0.80510276 | NEUTRAL | 0.54808806 |
| V | 107 | I | NEUTRAL | 0.66524217 | DELETERIOUS | 0.74451098 |
| F | 108 | V | DELETERIOUS | 0.80510276 | DELETERIOUS | 0.69 |
| R | 109 | K | DELETERIOUS | 0.72038776 | NEUTRAL | 0.47222222 |
| I | 112 | V | NEUTRAL | 0.55439739 | DELETERIOUS | 0.71856287 |
| I | 113 | S | DELETERIOUS | 0.72038776 | DELETERIOUS | 0.69860279 |
| I | 113 | T | NEUTRAL | 0.58359402 | NEUTRAL | 0.65005794 |
| I | 117 | V | NEUTRAL | 0.66524217 | DELETERIOUS | 0.78 |
| R | 119 | W | DELETERIOUS | 0.62208185 | DELETERIOUS | 0.61 |
| R | 119 | Q | DELETERIOUS | 0.62208185 | NEUTRAL | 0.64542294 |
| S | 122 | N | NEUTRAL | 0.70878378 | NEUTRAL | 0.56481481 |
| G | 123 | R | DELETERIOUS | 0.55551884 | NEUTRAL | 0.64542294 |
| V | 125 | L | NEUTRAL | 0.66524217 | DELETERIOUS | 0.65269461 |
| P | 127 | T | NEUTRAL | 0.50014676 | NEUTRAL | 0.64351852 |
| I | 130 | V | NEUTRAL | 0.66524217 | DELETERIOUS | 0.734 |
| I | 130 | T | DELETERIOUS | 0.72038776 | DELETERIOUS | 0.744 |
| D | 137 | N | DELETERIOUS | 0.80510276 | DELETERIOUS | 0.662 |
| T | 142 | I | NEUTRAL | 0.66524217 | NEUTRAL | 0.6400463 |
| P | 147 | S | NEUTRAL | 0.70878378 | DELETERIOUS | 0.56686627 |
| P | 149 | L | NEUTRAL | 0.55439739 | NEUTRAL | 0.48611111 |
| P | 149 | T | NEUTRAL | 0.55439739 | DELETERIOUS | 0.76 |
| G | 150 | R | DELETERIOUS | 0.72038776 | DELETERIOUS | 0.71856287 |
| V | 152 | G | NEUTRAL | 0.50014676 | NEUTRAL | 0.55671296 |
| E | 153 | Q | NEUTRAL | 0.70878378 | DELETERIOUS | 0.71457086 |
| I | 154 | R | DELETERIOUS | 0.72038776 | NEUTRAL | 0.56199305 |
| P | 158 | S | NEUTRAL | 0.66524217 | DELETERIOUS | 0.744 |
| D | 160 | Y | DELETERIOUS | 0.62208185 | DELETERIOUS | 0.71457086 |
| G | 161 | R | DELETERIOUS | 0.72038776 | NEUTRAL | 0.65005794 |
| T | 162 | S | NEUTRAL | 0.83058824 | NEUTRAL | 0.48319815 |
| T | 166 | I | NEUTRAL | 0.61167883 | UNKNOWN | 0 |
| E | 174 | D | NEUTRAL | 0.83058824 | NEUTRAL | 0.54808806 |
| G | 175 | D | NEUTRAL | 0.61167883 | DELETERIOUS | 0.78 |
| G | 177 | D | DELETERIOUS | 0.72038776 | NEUTRAL | 0.56481481 |
| V | 178 | I | NEUTRAL | 0.66524217 | NEUTRAL | 0.47222222 |
| M | 180 | V | NEUTRAL | 0.58359402 | NEUTRAL | 0.67091541 |
| M | 180 | L | NEUTRAL | 0.66524217 | DELETERIOUS | 0.56686627 |
| G | 181 | V | NEUTRAL | 0.58359402 | DELETERIOUS | 0.66067864 |
| M | 182 | I | NEUTRAL | 0.55439739 | DELETERIOUS | 0.56686627 |
| Y | 183 | H | NEUTRAL | 0.61167883 | DELETERIOUS | 0.84231537 |
| Y | 183 | C | DELETERIOUS | 0.80510276 | NEUTRAL | 0.47222222 |
| I | 189 | V | NEUTRAL | 0.61167883 | NEUTRAL | 0.69907407 |
| E | 190 | K | NEUTRAL | 0.66524217 | DELETERIOUS | 0.734 |
| A | 193 | T | DELETERIOUS | 0.72038776 | DELETERIOUS | 0.61077844 |
| H | 194 | Y | NEUTRAL | 0.61167883 | NEUTRAL | 0.56365741 |
| Q | 198 | R | NEUTRAL | 0.66524217 | NEUTRAL | 0.48032407 |
| A | 200 | S | NEUTRAL | 0.61167883 | NEUTRAL | 0.48319815 |
| L | 201 | V | NEUTRAL | 0.70878378 | NEUTRAL | 0.66550926 |
| S | 202 | A | NEUTRAL | 0.83058824 | NEUTRAL | 0.63541667 |
| K | 203 | R | NEUTRAL | 0.70878378 | NEUTRAL | 0.56481481 |
| G | 204 | V | NEUTRAL | 0.50014676 | DELETERIOUS | 0.78 |
| L | 207 | W | DELETERIOUS | 0.62208185 | DELETERIOUS | 0.734 |
| Y | 208 | H | DELETERIOUS | 0.80510276 | DELETERIOUS | 0.78 |
| Y | 208 | C | DELETERIOUS | 0.72038776 | DELETERIOUS | 0.68662675 |
| T | 214 | S | DELETERIOUS | 0.72038776 | DELETERIOUS | 0.68662675 |
| K | 217 | R | NEUTRAL | 0.50014676 | DELETERIOUS | 0.76 |
| Y | 219 | H | DELETERIOUS | 0.80510276 | DELETERIOUS | 0.84231537 |
| Y | 219 | C | DELETERIOUS | 0.84845361 | DELETERIOUS | 0.71856287 |
| D | 220 | G | DELETERIOUS | 0.72038776 | DELETERIOUS | 0.654 |
| R | 222 | H | NEUTRAL | 0.50014676 | DELETERIOUS | 0.74451098 |
| R | 222 | C | DELETERIOUS | 0.55551884 | DELETERIOUS | 0.654 |
| E | 229 | V | DELETERIOUS | 0.55551884 | DELETERIOUS | 0.68662675 |
| Y | 231 | H | DELETERIOUS | 0.62208185 | NEUTRAL | 0.56365741 |
| D | 232 | N | NEUTRAL | 0.76823399 | DELETERIOUS | 0.654 |
| K | 233 | M | NEUTRAL | 0.61167883 | DELETERIOUS | 0.74451098 |
| Y | 235 | C | DELETERIOUS | 0.72038776 | NEUTRAL | 0.47222222 |
| K | 236 | Q | NEUTRAL | 0.55439739 | NEUTRAL | 0.54808806 |
| Q | 238 | H | DELETERIOUS | 0.55551884 | NEUTRAL | 0.48435689 |
| F | 239 | L | DELETERIOUS | 0.55551884 | NEUTRAL | 0.63310185 |
| Q | 242 | H | NEUTRAL | 0.76823399 | NEUTRAL | 0.56365741 |
| K | 243 | E | NEUTRAL | 0.61167883 | NEUTRAL | 0.48611111 |
| K | 243 | R | NEUTRAL | 0.55439739 | DELETERIOUS | 0.76 |
| Y | 246 | H | DELETERIOUS | 0.84845361 | DELETERIOUS | 0.744 |
| D | 252 | N | DELETERIOUS | 0.88519637 | DELETERIOUS | 0.74251497 |
| A | 256 | V | DELETERIOUS | 0.62208185 | DELETERIOUS | 0.678 |
| A | 256 | S | NEUTRAL | 0.55439739 | DELETERIOUS | 0.734 |
| K | 260 | N | DELETERIOUS | 0.62208185 | DELETERIOUS | 0.78 |
| G | 263 | E | DELETERIOUS | 0.62208185 | NEUTRAL | 0.47222222 |
| G | 264 | A | NEUTRAL | 0.61167883 | NEUTRAL | 0.64542294 |
| I | 266 | V | NEUTRAL | 0.66524217 | DELETERIOUS | 0.71856287 |
| D | 273 | G | DELETERIOUS | 0.84845361 | DELETERIOUS | 0.76 |
| G | 274 | S | DELETERIOUS | 0.88519637 | DELETERIOUS | 0.71457086 |
| V | 276 | M | NEUTRAL | 0.50014676 | DELETERIOUS | 0.76 |
| S | 278 | P | DELETERIOUS | 0.84845361 | DELETERIOUS | 0.76646707 |
| S | 278 | L | DELETERIOUS | 0.62208185 | DELETERIOUS | 0.78 |
| D | 279 | H | DELETERIOUS | 0.84845361 | NEUTRAL | 0.54808806 |
| S | 280 | C | NEUTRAL | 0.76823399 | NEUTRAL | 0.65005794 |
| S | 280 | F | NEUTRAL | 0.70878378 | DELETERIOUS | 0.68662675 |
| M | 291 | T | DELETERIOUS | 0.55551884 | DELETERIOUS | 0.74251497 |
| T | 292 | I | DELETERIOUS | 0.62208185 | DELETERIOUS | 0.74451098 |
| S | 293 | I | DELETERIOUS | 0.84845361 | DELETERIOUS | 0.678 |
| V | 294 | M | NEUTRAL | 0.55439739 | DELETERIOUS | 0.71856287 |
| P | 298 | L | DELETERIOUS | 0.62208185 | DELETERIOUS | 0.744 |
| G | 300 | V | DELETERIOUS | 0.72038776 | DELETERIOUS | 0.568 |
| G | 300 | D | NEUTRAL | 0.50014676 | NEUTRAL | 0.48611111 |
| T | 302 | A | NEUTRAL | 0.66524217 | DELETERIOUS | 0.67065868 |
| E | 304 | G | DELETERIOUS | 0.80510276 | NEUTRAL | 0.56199305 |
| A | 305 | T | NEUTRAL | 0.66524217 | DELETERIOUS | 0.678 |
| E | 306 | A | DELETERIOUS | 0.84845361 | DELETERIOUS | 0.66067864 |
| H | 309 | R | DELETERIOUS | 0.88519637 | DELETERIOUS | 0.678 |
| H | 309 | Q | DELETERIOUS | 0.88519637 | DELETERIOUS | 0.78 |
| G | 310 | R | DELETERIOUS | 0.84845361 | DELETERIOUS | 0.744 |
| R | 314 | H | DELETERIOUS | 0.80510276 | DELETERIOUS | 0.84231537 |
| R | 314 | C | DELETERIOUS | 0.84845361 | DELETERIOUS | 0.68662675 |
| H | 315 | D | DELETERIOUS | 0.88519637 | DELETERIOUS | 0.71856287 |
| R | 317 | H | DELETERIOUS | 0.62208185 | DELETERIOUS | 0.74451098 |
| R | 317 | C | DELETERIOUS | 0.84845361 | DELETERIOUS | 0.65269461 |
| R | 317 | L | DELETERIOUS | 0.80510276 | NEUTRAL | 0.66975666 |
| M | 318 | T | NEUTRAL | 0.50014676 | DELETERIOUS | 0.56686627 |
| K | 321 | E | NEUTRAL | 0.55439739 | DELETERIOUS | 0.78 |
| T | 325 | M | DELETERIOUS | 0.72038776 | DELETERIOUS | 0.74251497 |
| S | 326 | P | NEUTRAL | 0.61167883 | DELETERIOUS | 0.76 |
| T | 327 | I | NEUTRAL | 0.50014676 | DELETERIOUS | 0.744 |
| N | 328 | S | DELETERIOUS | 0.72038776 | DELETERIOUS | 0.74451098 |
| N | 328 | K | DELETERIOUS | 0.84845361 | DELETERIOUS | 0.61 |
| I | 330 | T | DELETERIOUS | 0.55551884 | DELETERIOUS | 0.76 |
| I | 333 | S | DELETERIOUS | 0.72038776 | DELETERIOUS | 0.69 |
| A | 335 | S | NEUTRAL | 0.58359402 | DELETERIOUS | 0.568 |
| R | 338 | S | NEUTRAL | 0.55439739 | DELETERIOUS | 0.76 |
| G | 339 | R | DELETERIOUS | 0.72038776 | NEUTRAL | 0.57242178 |
| A | 341 | V | NEUTRAL | 0.61167883 | NEUTRAL | 0.47222222 |
| A | 341 | P | NEUTRAL | 0.58359402 | NEUTRAL | 0.56365741 |
| A | 341 | D | NEUTRAL | 0.58359402 | DELETERIOUS | 0.78 |
| L | 346 | P | DELETERIOUS | 0.62208185 | NEUTRAL | 0.48435689 |
| E | 351 | G | NEUTRAL | 0.50014676 | NEUTRAL | 0.64542294 |
| E | 351 | K | NEUTRAL | 0.70878378 | NEUTRAL | 0.56365741 |
| A | 353 | G | NEUTRAL | 0.76823399 | NEUTRAL | 0.56365741 |
| A | 353 | D | NEUTRAL | 0.70878378 | NEUTRAL | 0.69907407 |
| F | 354 | V | NEUTRAL | 0.76823399 | NEUTRAL | 0.69907407 |
| F | 354 | L | NEUTRAL | 0.76823399 | NEUTRAL | 0.48435689 |
| A | 356 | V | NEUTRAL | 0.61167883 | NEUTRAL | 0.69907407 |
| S | 363 | A | NEUTRAL | 0.76823399 | NEUTRAL | 0.69907407 |
| I | 364 | V | NEUTRAL | 0.76823399 | NEUTRAL | 0.61645423 |
| I | 364 | L | NEUTRAL | 0.76823399 | DELETERIOUS | 0.61 |
| E | 365 | V | NEUTRAL | 0.58359402 | NEUTRAL | 0.68055556 |
| I | 367 | V | NEUTRAL | 0.76823399 | DELETERIOUS | 0.66067864 |
| I | 367 | T | DELETERIOUS | 0.55551884 | NEUTRAL | 0.54808806 |
| A | 369 | V | NEUTRAL | 0.61167883 | DELETERIOUS | 0.74451098 |
| G | 370 | V | DELETERIOUS | 0.72038776 | DELETERIOUS | 0.734 |
| M | 372 | T | DELETERIOUS | 0.72038776 | DELETERIOUS | 0.74451098 |
| T | 373 | I | DELETERIOUS | 0.72038776 | DELETERIOUS | 0.744 |
| T | 373 | N | DELETERIOUS | 0.80510276 | DELETERIOUS | 0.678 |
| K | 374 | E | NEUTRAL | 0.58359402 | NEUTRAL | 0.67091541 |
| A | 378 | V | NEUTRAL | 0.76823399 | NEUTRAL | 0.69907407 |
| A | 378 | G | NEUTRAL | 0.76823399 | NEUTRAL | 0.55671296 |
| L | 383 | F | NEUTRAL | 0.55439739 | NEUTRAL | 0.68055556 |
| P | 384 | L | NEUTRAL | 0.61167883 | NEUTRAL | 0.57242178 |
| N | 385 | S | NEUTRAL | 0.61167883 | NEUTRAL | 0.64351852 |
| N | 385 | D | NEUTRAL | 0.70878378 | DELETERIOUS | 0.56686627 |
| R | 388 | H | NEUTRAL | 0.50014676 | DELETERIOUS | 0.71457086 |
| R | 388 | C | NEUTRAL | 0.50014676 | NEUTRAL | 0.64542294 |
| M | 398 | I | NEUTRAL | 0.76823399 | DELETERIOUS | 0.678 |
| D | 399 | H | NEUTRAL | 0.50014676 | DELETERIOUS | 0.66067864 |
| D | 399 | G | DELETERIOUS | 0.55551884 | DELETERIOUS | 0.71457086 |
| L | 401 | P | DELETERIOUS | 0.62208185 | NEUTRAL | 0.63541667 |
| G | 402 | E | NEUTRAL | 0.76823399 | DELETERIOUS | 0.67065868 |
| L | 405 | V | NEUTRAL | 0.58359402 | NEUTRAL | 0.65005794 |
| K | 408 | Q | NEUTRAL | 0.70878378 | NEUTRAL | 0.48435689 |
